# MOGAD patient autoantibodies induce complement, phagocytosis, and cellular cytotoxicity

**DOI:** 10.1172/jci.insight.165373

**Published:** 2023-06-08

**Authors:** Soumya S. Yandamuri, Beata Filipek, Abeer H. Obaid, Nikhil Lele, Joshua M. Thurman, Naila Makhani, Richard J. Nowak, Yong Guo, Claudia F. Lucchinetti, Eoin P. Flanagan, Erin E. Longbrake, Kevin C. O’Connor

**Affiliations:** 1 Department of Neurology and; 2 Department of Immunobiology, Yale School of Medicine, New Haven, Connecticut, USA.; 3 Department of Pharmaceutical Microbiology and Biochemistry, Medical University of Lodz, Lodz, Poland.; 4 Institute of Biomedical Studies, Baylor University, Waco, Texas, USA.; 5 Department of Medicine, University of Colorado School of Medicine, Anschutz Medical Campus, Aurora, Colorado, USA.; 6 Department of Pediatrics, Yale School of Medicine, New Haven, Connecticut, USA.; 7 Department of Neurology and Center for MS and Autoimmune Neurology, Mayo Clinic, Rochester, Minnesota, USA.

**Keywords:** Autoimmunity, Neuroscience, Autoimmune diseases, Demyelinating disorders, Neurological disorders

## Abstract

Myelin oligodendrocyte glycoprotein (MOG) antibody–associated disease (MOGAD) is an inflammatory demyelinating CNS condition characterized by the presence of MOG autoantibodies. We sought to investigate whether human MOG autoantibodies are capable of mediating damage to MOG-expressing cells through multiple mechanisms. We developed high-throughput assays to measure complement activity (CA), complement-dependent cytotoxicity (CDC), antibody-dependent cellular phagocytosis (ADCP), and antibody-dependent cellular cytotoxicity (ADCC) of live MOG-expressing cells. MOGAD patient sera effectively mediate all of these effector functions. Our collective analyses reveal that (a) cytotoxicity is not incumbent on MOG autoantibody quantity alone; (b) engagement of effector functions by MOGAD patient serum is bimodal, with some sera exhibiting cytotoxic capacity while others did not; (c) the magnitude of CDC and ADCP is elevated closer to relapse, while MOG-IgG binding is not; and (d) all IgG subclasses can damage MOG-expressing cells. Histopathology from a representative MOGAD case revealed congruence between lesion histology and serum CDC and ADCP, and we identified NK cells, mediators of ADCC, in the cerebrospinal fluid of relapsing patients with MOGAD. Thus, MOGAD-derived autoantibodies are cytotoxic to MOG-expressing cells through multiple mechanisms, and assays quantifying CDC and ADCP may prove to be effective tools for predicting risk of future relapses.

## Graphical abstract

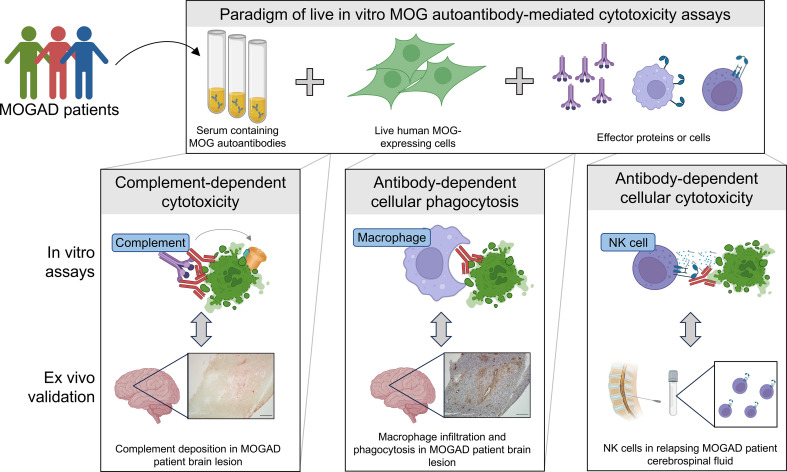

## Introduction

Myelin oligodendrocyte glycoprotein (MOG) antibody–associated disease (MOGAD) is a disease entity defined by the presence of circulating MOG autoantibodies. The core clinical spectrum of MOGAD includes optic neuritis, myelitis, and acute demyelinating encephalomyelitis (ADEM), with additional clinical phenotypes continuing to emerge (1). Because MOG is expressed on the surface of the myelin sheath, it is presumed to be easily accessible to antibodies, cells, and other immune system components and has been studied for years as a potential autoimmune target in multiple sclerosis (MS) and other autoimmune neurologic diseases. Early attempts to identify MOG autoantibodies using ELISAs led to the conclusion that these antibodies were nonspecific and nonpathologic, since a proportion of the general population harbors antibodies to the extracellular linear domain of MOG (2, 3). However, when a conformationally correct and glycosylated human MOG was used to identify MOG autoantibodies in serum and CSF, they were not found in patients with MS but were rather found in about 40% of patients diagnosed with ADEM or aquaporin 4 IgG–negative (AQP4-IgG^–^) neuromyelitis optica spectrum disorder (NMOSD) (3–7). These findings led to the recategorization of MOGAD as a separate disease entity.

Thus, prior to the development and availability of accurate MOG autoantibody testing, many individuals who would now be diagnosed with MOGAD were categorized within the clinical spectrum of NMOSD (6, 8, 9). Approximately 70% of patients with NMOSD harbor circulating IgG autoantibodies to AQP4 (10–12), a water channel on the end-feet of astrocytes (13–15). IgG1 antibodies, the predominant subclass of AQP4-IgG in NMOSD (13), mediate a variety of Fc-dependent effector functions, including complement-dependent cytotoxicity (CDC) and antibody-dependent cellular cytotoxicity (ADCC), a mechanism by which NK cells eliminate antibody-bound cells. AQP4-IgG are pathogenic in vitro and in mice through these effector functions (16–20). These mechanistic insights implicated autoantibodies and CDC as pathogenic. Clinical trials subsequently confirmed that B cell depletion and complement inhibition effectively suppress clinical relapses, and 3 therapeutics based on these mechanisms were subsequently approved for the treatment of AQP4-IgG^+^ NMOSD (21–25).

Despite similarities with AQP4-IgG^+^ NMOSD, patients with MOGAD are more likely to develop simultaneous bilateral optic neuritis, exhibit better outcomes and clinical recovery, and experience less frequent relapses (7, 26, 27). Distinctions between MOGAD and NMOSD also exist at the cell level. Histology reveals relative sparing of both astrocytes and oligodendrocytes in MOGAD lesions, despite demyelination (28). Importantly, in both MOGAD and NMOSD, disability appears to accumulate during relapse (29–31), typically consisting of varying visual, motor, ambulatory, bladder, bowel, and/or cognitive dysfunction (31, 32). Therefore, a primary focus of therapeutic intervention for both conditions is relapse prevention. Nevertheless, there are currently no approved treatments for MOGAD, and the pathophysiology of the disease is less completely characterized than for AQP4-IgG^+^ NMOSD. We hypothesized that adopting a similar strategy for elucidating pathophysiologic mechanisms may identify therapeutic targets and biomarkers.

Thus far, in vitro and in situ studies have demonstrated that human MOG autoantibodies are capable of mediating CDC (33–36). One study has shown that sera from pediatric patients with MOGAD are capable of inducing ADCC in vitro. Histopathology of demyelinating lesions in patients with MOGAD exhibit complement deposition as well as marked infiltration and activation of macrophages and microglia (28, 37). However, it is unclear if macrophages and microglia can directly damage live MOG-expressing cells through antibody-dependent cellular phagocytosis (ADCP), which is the engulfment of antibody-bound cells, or if they merely remove debris. While these studies imply that MOG autoantibodies have pathogenic potential, an enduring question is whether MOG autoantibodies are pathogenic or epiphenomena of disease. In this study, we sought to further investigate whether serum MOG autoantibodies are capable of multiple mechanisms of cytotoxicity. We developed high-throughput assays to quantify complement activity (CA), CDC, ADCC, and ADCP of live cells expressing human MOG. In order to evaluate the potential clinical value of these assays, we characterized the relationships between serum effector function and quantity of binding MOG-IgG, recapitulation of serum effector functions in CNS neuropathology, and correlation between effector functions and relapse.

## Results

### High-throughput MOG CDC and ADCP assays.

**Figure 1:**
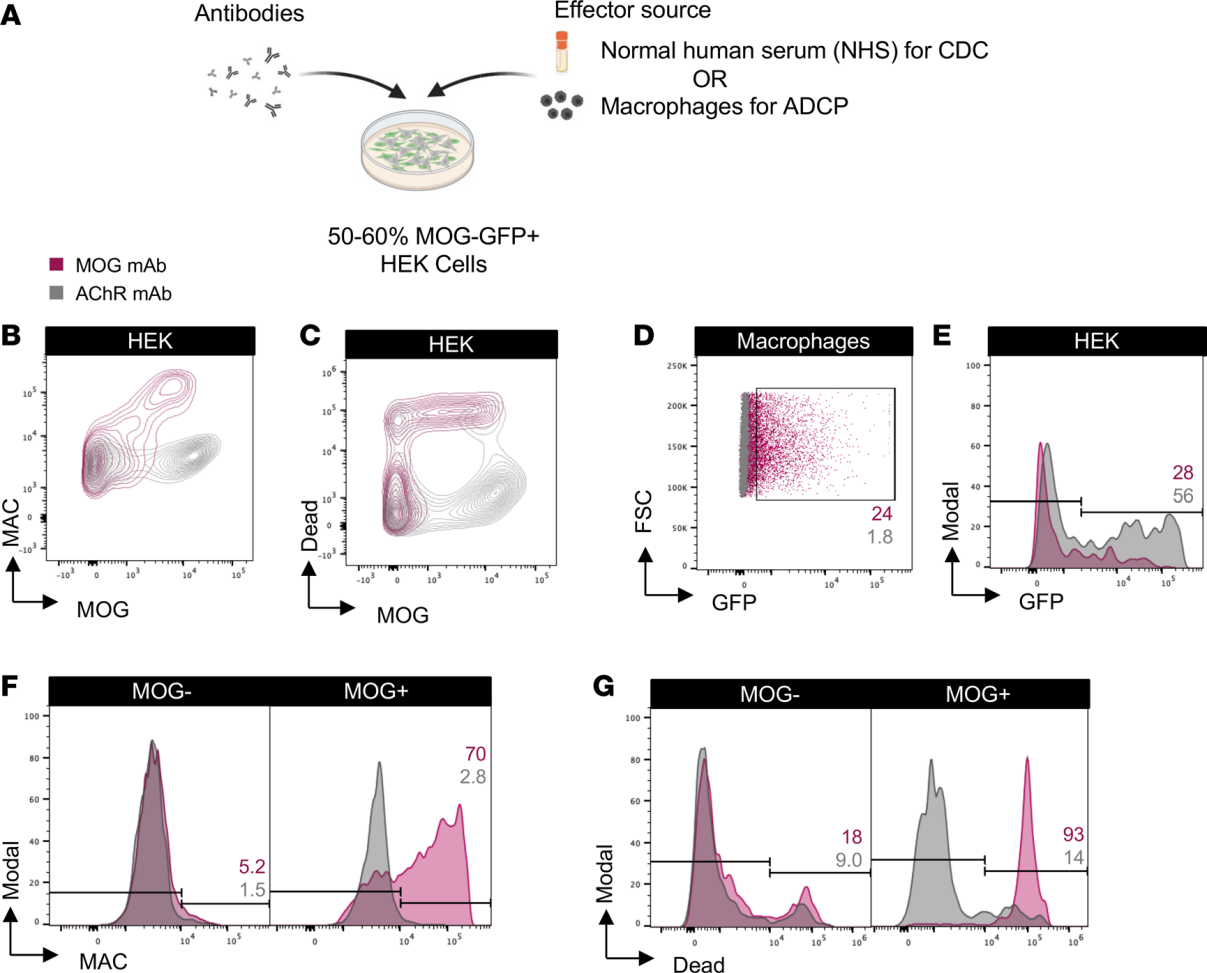
MOG IgG1 mAb induces CDC and ADCP of live MOG-expressing cells in vitro. (**A**) Schematic of CDC and ADCP assays utilizing live HEK cells partially transfected with full-length human MOG-GFP, incubated with 1 μg/mL MOG or control AChR mAbs, followed by the addition of NHS for CDC or macrophages for ADCP induction. MAC formation and cell death for CDC and phagocytosis and loss of MOG^+^ cells for ADCP were quantified by flow cytometry. (**B** and **C**) Contour plots depict (**B**) MAC formation and (**C**) death of HEK cells based on MOG expression upon incubation with MOG versus AChR mAbs in the CDC assay. (**D** and **E**) Macrophage phagocytosis of MOG^+^ cells is shown by (**D**) dot plots depicting the frequency of GFP^+^ macrophages, and (**E**) histograms of MOG^+^ cells out of the total HEK cell population, upon incubation with MOG versus AChR mAbs in the ADCP assay. (**F** and **G**) Histograms show (**F**) MAC formation and (**G**) death of MOG^–^ versus MOG^+^ HEK cells upon incubation with MOG versus AChR mAbs in the CDC assay. All graphs are representative. Each experiment was performed at least 3 times in duplicate. Frequencies of indicated gates depicted on plots.

We developed effector function assays modeled on flow cytometry cell-based assays (CBA) using live human embryonic kidney 293T (HEK) cells. The cells were transiently transfected to induce expression of full-length human MOG-GFP in its native conformation. Approximately 50%–60% of the HEK cells expressed MOG following transfection, providing the opportunity to observe effects on both MOG^+^ and MOG^–^ cells. The assays involved incubation of antibodies with the transfected HEK cells to allow for binding. Then, normal human serum (NHS), as a source of human complement, or THP-1 macrophages were added (Figure 1A). In the CDC assay, we observed marked membrane attack complex (MAC) formation and death of MOG^+^ cells (using a live/dead stain) in the presence of a MOG mAb (subclass of all mAbs is IgG1 unless otherwise specified) but not a control acetylcholine receptor (AChR) mAb (Figure 1, B and C). In the presence of the MOG mAb, the macrophages phagocytose MOG^+^ cells, as indicated by GFP in the macrophages (Figure 1D). Moreover, the frequency of MOG^+^ cells out of the total HEK cell population was diminished, demonstrating their elimination (Figure 1E). MAC deposition and death of MOG^+^ cells, but not MOG^–^ cells, further confirmed the MOG specificity of the CDC assay (Figure 1, F and G).

### MOG IgG1 and IgG3 subclass autoantibodies induce CDC, while all IgG subclasses are capable of ADCP.

**Figure 2:**
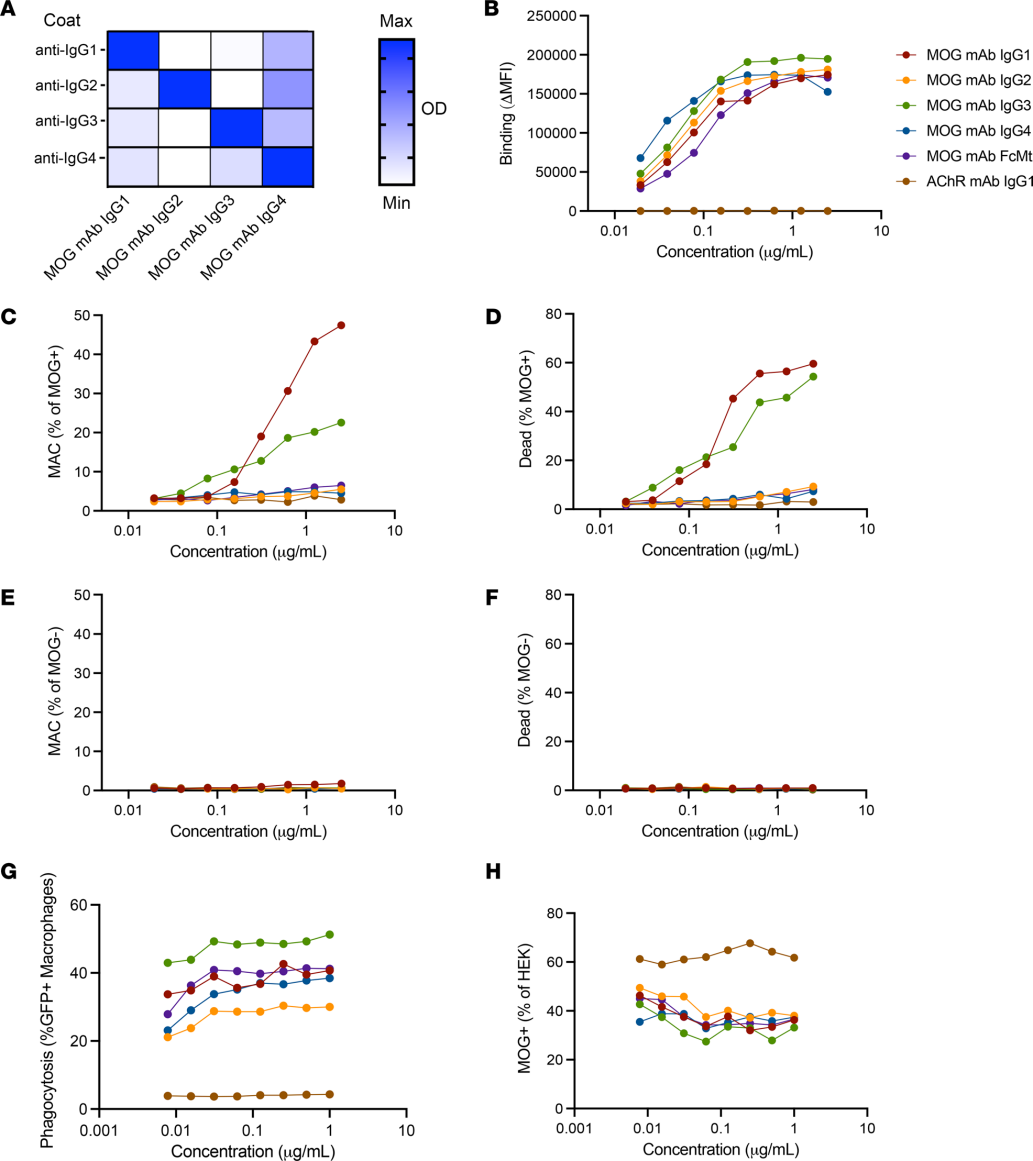
MOG IgG1 and IgG3 induce CDC while all IgG subclasses induce ADCP. The MOG mAb variable region was subcloned into Fc vectors to recombinantly produce MOG IgG1, IgG2, IgG3, IgG4, and Fc mutant (FcMt) mAbs. (**A**) Sandwich ELISAs indicate binding of MOG IgG1, IgG2, IgG3, and IgG4 mAbs at 10 μg/mL to commensurate subclass-specific antibodies. Serial dilutions of the 4 MOG subclass mAbs, the MOG FcMt mAb, and the AChR IgG1 mAb were tested for MOG binding and effector functions. (**B**) MAb binding to MOG was quantified as ΔMFI using a live flow cytometry MOG-CBA. (**C**–**F**) MAC formation and death of (**C** and **D**) MOG^+^ and (**E** and **F**) MOG^–^ cells in the CDC assay. (**G** and **H**) Phagocytosis and MOG^+^ cells out of total HEK cells in the ADCP assay. Each experiment was performed at least 2 times in duplicate. In **B**–**H**, each dot represents the average of duplicates.

While all MOG-IgG^+^ patients harbor MOG IgG1 antibodies, MOG IgG2, IgG3, and IgG4 antibodies have also been detected in some patients (38, 39). Considering that antibody Fc mediates effector functions, we were curious about the differential ability of the 4 IgG subclasses to mediate damage to MOG-expressing cells. Thus, we generated recombinant MOG mAbs with varied Fc by subcloning the variable region of the MOG mAb into IgG2, IgG3, and IgG4 subclass expression vectors as well as an IgG1 Fc mutant (FcMt) vector that cannot induce CDC or ADCC (40). We expressed and purified these mAbs and validated IgG subclass expression using sandwich ELISAs (Figure 2A). Then, we confirmed binding to MOG in a CBA. Binding was calculated as the difference (Δ) in mean fluorescence intensity (MFI) of IgG on MOG^+^ cells minus that of MOG^–^ cells (ΔMFI = MFI_MOG+_ – MFI_MOG–_), in order to eliminate the contribution of nonspecific IgG binding to HEK cells. When we performed the CBA with serial dilutions of the mAbs, all 5 MOG mAbs exhibited similar binding to MOG, while the AChR mAb did not (Figure 2B). As expected, the CDC assay showed that IgG1 and IgG3 MOG mAbs were capable of inducing CDC, both MAC deposition and death, of MOG-expressing cells, while MOG IgG2, IgG4, FcMt mAbs, and the AChR mAb were not (Figure 2, C and D). CDC induction by MOG IgG1 and IgG3 was specific for MOG; they did not induce MAC deposition or cell death of MOG^–^ cells (Figure 2, E and F). However, all 5 mAbs induced ADCP, including the FcMt that had abrogated CDC (Figure 2, G and H).

### MOGAD serum induces bimodal CDC and ADCP of live MOG-expressing cells.

**Figure 3:**
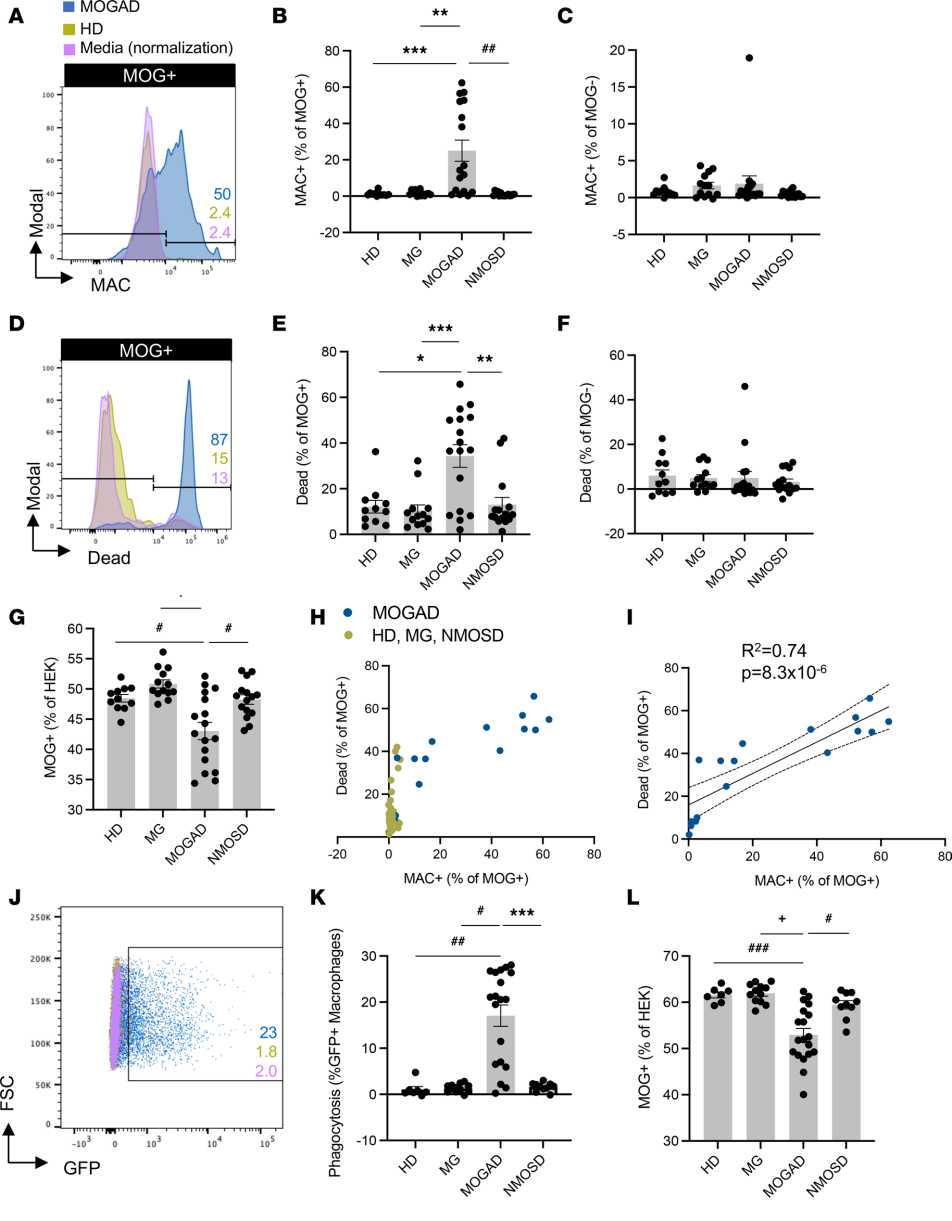
MOGAD patient serum induces CDC and ADCP of live MOG-expressing cells while HD, MG, and NMOSD serum do not.(A–L) HI serum from patients with MOGAD (*n*_CDC_ = 17, *n*_ADCP_ = 19), MG (*n*_CDC_ = 13, *n*_ADCP_ = 12), and NMOSD (*n*_CDC_ = 15, *n*_ADCP_ = 10) and HD (*n*_CDC_ = 11, *n*_ADCP_ = 7) were evaluated for CDC (**A**–**I**) and ADCP induction (**J**–**L**), normalized to that of media alone (no antibodies or donor serum). (**A**) Representative histograms depict MAC deposition on MOG^+^ cells by MOGAD versus HD serum in the CDC assay. (**B** and **C**) Comparative MAC formation on (**B**) MOG^+^ and (**C**) MOG^–^ cells by condition. (**D**) Representative histogram depicts dead MOG^+^ cells by MOGAD versus HD serum. (**E** and **F**) Comparative dead (**E**) MOG^+^ and (**F**) MOG^–^ cells by condition. (**G**) Resultant frequency of MOG^+^ cells out of total HEK cells. (**H**) Comparison of frequency of MAC formation versus death of MOG^+^ cells per sample. (**I**) Linear regression of MOGAD samples only (goodness of fit, *R*^2^, and significance of nonzero slope, *P* value, shown on graph). (**J**) Representative dot plot depicts frequency of phagocytosing macrophages (GFP^+^) upon incubation with MOGAD versus HD serum in ADCP assay. (**K** and **L**) Frequency of (**K**) phagocytosing macrophages and (**L**) MOG^+^ cells out of total HEK cells by condition. Each dot represents a patient (average of duplicates), normalized to media-only control, and bars depict mean ± SEM. Normality test followed by Kruskal-Wallis for **B** (*P* = 2.3 ***×*** 10^–4^), **C** (*P* = 0.22), **E** (*P* = 5.6 ***×*** 10^–3^), and **K** (*P* = 1.4 ***×*** 10^–5^) and 1-way ANOVA for **G** (*P* = 1.2 ***×*** 10^–5^) and **L** (*P* = 1.1 ***×*** 10^–5^). For *P* ≤ 0.05, multiple comparisons were corrected with FDR of 0.05 and depicted on graph (*P ≤ 0.05, **P ≤ 0.01, ***P ≤ 0.005, ^#^*P* ≤ 0.0005, ^##^*P* ≤ 0.0001, ^###^*P* ≤ 0.00005, ^+^*P* ≤ 0.00001).

After confirming the functional performance of the assays using mAbs, we then evaluated the ability of MOGAD patient serum to induce these effector functions. All serum samples were heat inactivated (HI) to abolish activity by endogenous complement proteins, allowing for assessment of autoantibody function only. We assessed CDC in a cohort of 17 clinically diagnosed patients with MOGAD, 11 healthy donors (HD), and autoimmune neurologic disease controls consisting of 15 patients with NMOSD and 13 with myasthenia gravis (MG) (summary cohort characteristics in Table 1, detailed MOGAD patient characteristics in Supplemental Table 1; supplemental material available online with this article; https://doi.org/10.1172/jci.insight.165373DS1). Serum from patients with MOGAD mediated MOG-specific complement deposition, while control serum did not (Figure 3, A and B). Specifically, MOGAD serum induced MAC formation on 25% (mean, normalized to media alone) of the MOG^+^ population, significantly more than HD (mean 1.2%), MG (mean 1.8%), and NMOSD (mean 0.93%) serum. No difference in the frequency of MAC^+^MOG^–^ cells between the conditions was observed (Figure 3C), indicating MOG-specific complement deposition; however, we did identify an outlier MOGAD sample that induced MAC formation on 19% of the MOG^–^ cell population.

Similarly, CDC of MOG^+^ cells was mediated by MOGAD serum but not control serum (Figure 3D). MOGAD serum induced a mean 34% MOG^+^ cell death, significantly more than HD (mean 12%), MG (mean 10%), or NMOSD (mean 13%) serum (Figure 3E). No difference in CDC was observed among MOG^–^ cells (Figure 3F). However, the same sample that induced MAC formation on MOG^–^ cells caused 46% MOG^–^ cell death. As a result of directed MOG^+^ cell death by CDC, the frequency of MOG^+^ cells out of total HEK cells was also reduced by MOGAD serum (Figure 3G). We then evaluated the relationship between MAC formation and cell death (Figure 3H) and found that linear regression on the MOGAD cohort fit a slope of 0.74 with an *R*^2^ of 0.74 (*P* = 8.3 ***×*** 10^–6^), demonstrating a positive association (Figure 3I). Collectively, we observed heterogeneity in the extent of CDC induced by MOGAD serum, both MAC deposition and cell death. A bimodal distribution for both metrics was observed; for example, 12 of 18 (67%) MOGAD serum samples induced robust MOG^+^ MAC deposition, while 6 of 18 (33%) induced negligible MOG^+^ MAC deposition with similar values as the controls. The signal/noise ratio (SNR) of MOGAD/HD serum CDC was 14 for MAC^+^MOG^+^ and 2.0 for dead MOG^+^ detection. When factor B–depleted NHS was used as the complement source to induce the classical complement cascade while preventing the alternative pathway, MOGAD patient serum still mediated MAC deposition and death of MOG^+^ cells, unlike control sera (Supplemental Figure 1, A and B). When a complement source was omitted, MAC deposition and CDC were not detected, exemplifying that the assays capture autoantibody characteristics alone (Supplemental Figure 1, C and D).

In the ADCP assay, we observed that MOGAD patient serum induced phagocytosis of MOG^+^ cells, while HD serum did not (Figure 3J). We assayed ADCP in a cohort of 19 MOGAD, 7 HD, 10 NMOSD, and 12 MG participants (Table 2 and Supplemental Table 1), the majority of whom overlapped with the CDC cohort. We observed ADCP by 17% (mean) of macrophages with MOGAD serum, and that was significantly more than HD (mean 1.1%), MG (mean 1.3%), or NMOSD (mean 1.6%) serum (Figure 3K). As a result, ADCP resulted in a reduction in the percentage of MOG^+^ cells out of total HEK cells (Figure 3L). Notably, while MOGAD serum-induced phagocytosis exhibited bimodal distribution, the resultant fraction of MOG^+^ cells was normally distributed. The SNR of MOGAD to HD serum ADCP was 5.5 for GFP^+^ THP-1 and 4.3 for frequency of MOG^+^ HEK detection. Collectively, these data show that MOGAD serum autoantibodies are capable of both CDC and ADCP, and these mechanisms specifically destroy cells that express MOG and spare those that do not.

### MOGAD patient serum effector functions recapitulate neuropathology.

**Figure 4:**
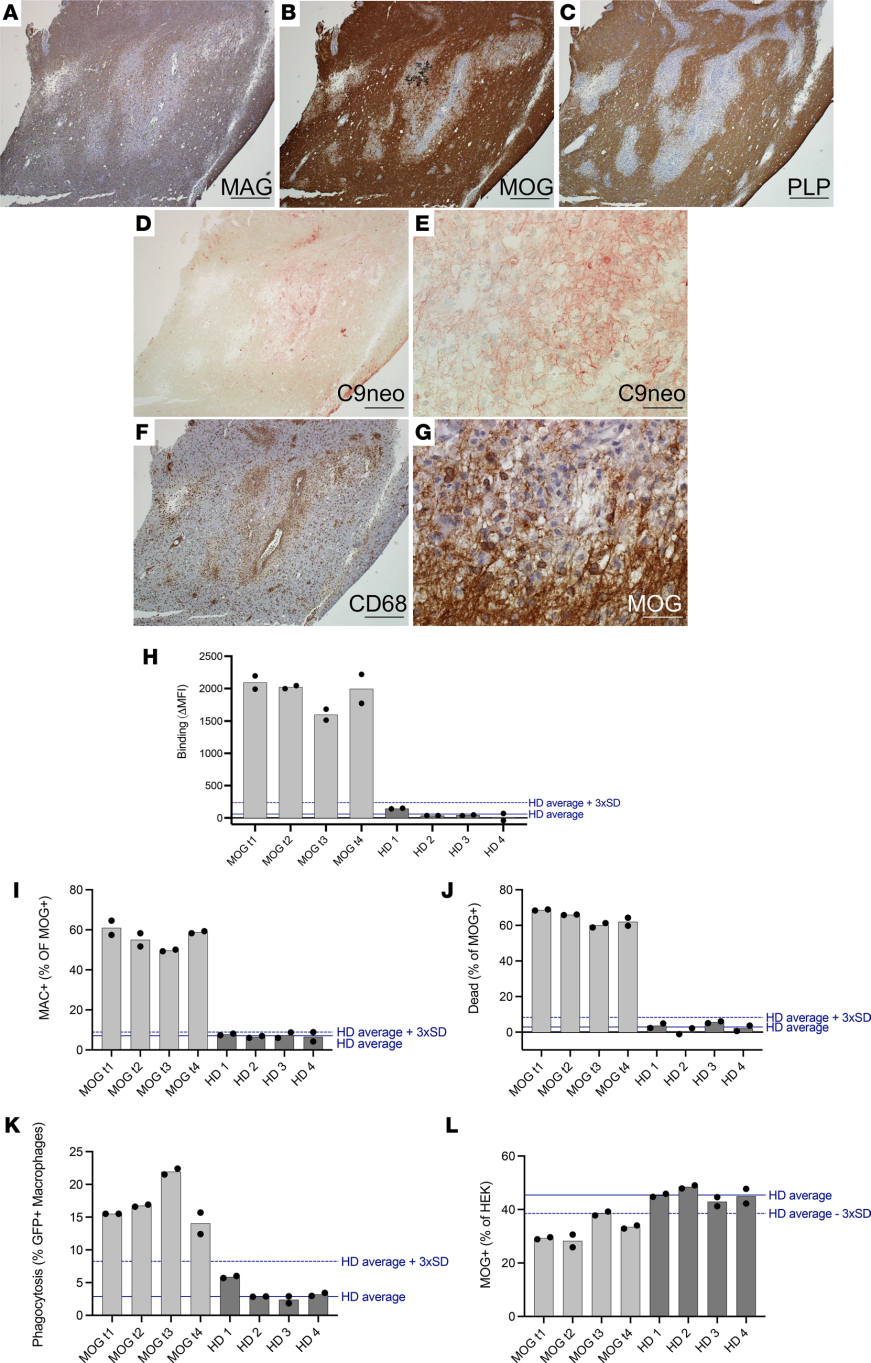
Neuropathology in frontal lobe biopsy of patient with MOGAD with paired serum effector functions. Right frontal lobe biopsy was undertaken in a symptomatic patient with MOGAD based on MRI findings. (**A**–**C**) Histology was performed and indicated active demyelinating lesions with loss of (**A**) MAG, (**B**) MOG, and (**C**) PLP. (**D** and **E**) Complement deposition in lesions indicated by (**D**) C9neo (red), with higher magnification on right (**E**). (**F** and **G**) CD68^+^ (brown) macrophage/microglia infiltration detected in lesions and (**G**) macrophages appear foamy and myelin-laden upon higher magnification of MOG staining. Scale bar: 500 μm (**A**–**D** and **F**) and 50 μm (**E** and **G**). (**H**) The patient’s serum was collected at 4 time points: during relapse (MOG t1), 2 days thereafter (MOG t2), and twice during remission (MOG t3, t4). The serum was tested for MOG binding IgG in comparison to serum from 4 HD in a live MOG-CBA. These samples were then tested for induction of CDC and ADCP effector functions. (**I**–**L**) Resultant (**I**) MAC formation and (**J**) dead MOG^+^ cells in CDC assay and (**K**) phagocytosis and (**L**) MOG^+^ out of total HEK cells in ADCP assay. Experiments shown in **H**–**L** were performed in duplicate, shown as dots, with bar showing their mean.

We next investigated whether effector mechanisms of serum biospecimens, in our in vitro assays, reflect neuropathological findings in a relapsing MOGAD case. A man in his 40s initially developed an upper respiratory tract infection followed by subacute onset of ADEM that progressed to coma and severe quadriparesis, requiring intubation and mechanical ventilation within 1 month. Diagnostic CSF and MRI findings can be found in Supplemental Figure 2. After 1 day of i.v. methylpredisone, a biopsy of the right frontal lobe was undertaken (Supplemental Figure 2A, arrowhead). Histology of the biopsy revealed active white matter demyelination, with loss of myelin-associated glycoprotein (MAG; Figure 4A), MOG (Figure 4B), and proteolipid protein (PLP; Figure 4C). C9 neoantigen (C9neo; Figure 4, D and E), marked CD68^+^ macrophage/microglia infiltration (Figure 4F), and myelin-laden macrophages (Figure 4G) illustrated complement deposition and phagocytosis. Subsequently, the patient’s serum tested positive for MOG-IgG in a live CBA at a high titer of 1:1,000 (normal < 1:20). After 3 months, the patient returned to normal, for the most part, with mild residual erectile, bladder, and bowel sequelae. However, 15 months following initial disease onset, the patient experienced a relapse consisting of bilateral optic neuritis and recovered after i.v. methylprednisone treatment. His MOG-IgG has remained persistently positive at high titer (1:100). Over this course, a total of 4 serum samples were taken, 2 in proximity to the first attack and 2 during remission following the second attack (Table 3). CDC and ADCP assays were performed on these serum samples along with 4 HD serum samples (50% male, mean age 41, SD 13). All 4 serum samples from the patient with MOGAD exhibited high levels of MOG-IgG (Figure 4H), MAC deposition on 50%–61%, and death of 60%–69% MOG^+^ cells in the CDC assay (Figure 4, I and J), 14%–22% macrophages that phagocytosed MOG^+^ cells, and a resultant loss in MOG^+^ cells in the ADCP assay (Figure 4, K and L). Thus, all 4 samples were capable of robust CDC and ADCP of MOG-expressing cells. These data suggest that myelin phagocytosis by infiltrating macrophages and microglia in lesions may reflect autoantibody-directed destruction of MOG-expressing cells. Moreover, serum autoantibody effector function assays may recapitulate pathology at the site of disease.

### Magnitude of CDC and ADCP correlate with MOG-IgG.

**Figure 5:**
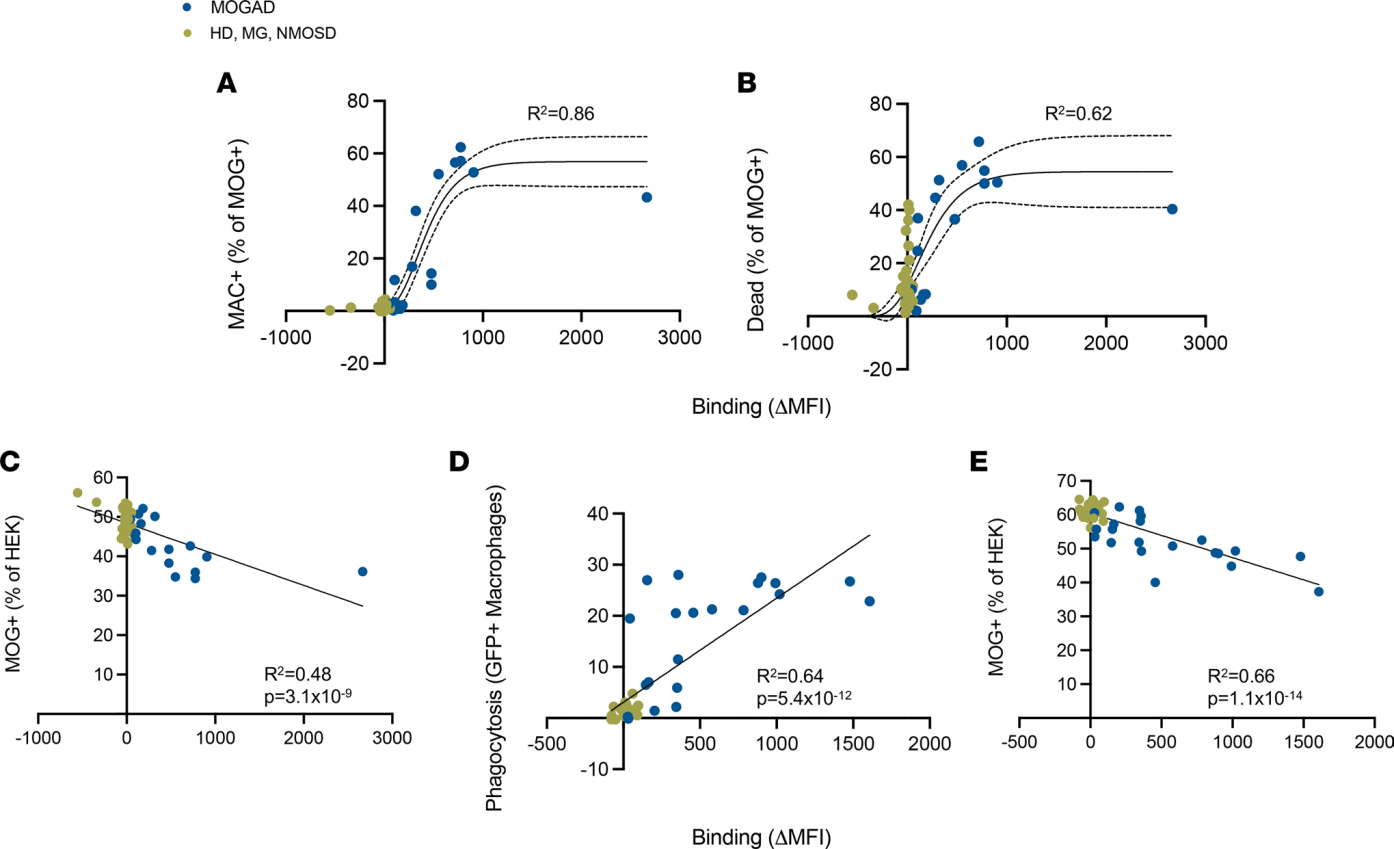
Magnitude of effector functions is associated with MOG-binding IgG in serum. A live MOG-CBA was used to quantify serum MOG-binding IgG and compared with CDC and ADCP induction of HI serum from patients with MOGAD (*n*_CDC_ = 17, *n*_ADCP_ = 19), MG (*n*_CDC_ = 13, *n*_ADCP_ = 12), and NMOSD (*n*_CDC_ = 15, *n*_ADCP_ = 10) and HD (*n*_CDC_ = 11, *n*_ADCP_ = 7). (**A** and **B**) MAC deposition and dead MOG^+^ cells upon CDC assay versus binding to MOG, fit with Gompertz model. (**C**) Frequency of MOG^+^ cells out of total HEK cells upon CDC assay versus binding to MOG, fit with linear regression model. (**D** and **E**) Phagocytosing macrophages and frequency of MOG^+^ cells out of total HEK cells upon ADCP assay versus binding to MOG, fit with linear regression model. Each dot represents a patient (average of duplicates). Gompertz models show 95% CI indicated by dotted lines. All models show goodness of fit, *R*^2^, on graph. Linear models show significance of nonzero slope, *P* value, on graph.

Given the congruence between serum autoantibody functions and neuropathology, we then investigated factors influencing effector functions. First, we employed CBA to quantify MOG-IgG in sera to assess their magnitude as a possible correlative factor. We observed that MAC formation only resulted from samples with a positive ΔMFI, as expected (Figure 5A). To determine the relationship between binding and MAC formation, we tested 4 nonlinear regression models and linear regression for fit, compared by Akaike’s Information Criterion (AICc) (41–43). Out of dose-response, 1-site specific binding, exponential plateau, Gompertz curve (44), and linear regression, the Gompertz curve fit best with an *R*^2^ of 0.86. We selected these models based on qualitative characteristics of the curves as well as antibody-antigen binding kinetics (45, 46). Death of MOG^+^ cells as a result of CDC was also best fit by the Gompertz curve with a *R*^2^ of 0.62 (Figure 5B). The Gompertz curve also best models these metrics for MOGAD samples only, when omitting control samples (Supplemental Figure 3, A and B).

The Gompertz model (44) suggests that a threshold of autoantibody binding to MOG-expressing cells must be exceeded for large relative increases in CDC; at lower autoantibody binding and at very high autoantibody binding, there is little difference in the change in CDC with changes in autoantibody binding. Linear regression models indicate positive associations between binding and MAC formation (*P* = 2.9 ***×*** 10^–6^) and binding and CDC (*P* = 3.6 ***×*** 10^–10^). Linear modeling was the best fit for the percentage of MOG^+^ cells out of total HEK after CDC but had low goodness of fit at *R*^2^ of 0.48 (Figure 5C). Nonetheless, binding was shown to induce a reduction in the frequency of MOG^+^ cells (*P* = 3.1 ***×*** 10^–9^). Linear models still exhibited a correlation when omitting control samples and evaluating MOGAD samples alone (Supplemental Figure 3C). Thus, despite interpatient heterogeneity, these regression analyses depict a correlation between the quantity of MOG-IgG and CDC.

The relationships between MOG-IgG and percentage of phagocytosing macrophages and of MOG^+^ cells, as a result of ADCP, were best modeled by linear regression, with fits of *R*^2^ = 0.64 and 0.66, respectively (Figure 5, D and E). They reveal associations between binding and phagocytosis (*P* = 5.4 ***×*** 10^–12^) and resultant loss in MOG^+^ cells (*P* = 1.1 ***×*** 10^–14^). These findings are recapitulated when evaluating the results of MOGAD patient serum samples alone without HD, MG, or NMOSD serum controls (Supplemental Figure 3, D and E).

As observed with CDC, there is interpatient heterogeneity in ADCP that cannot be attributed to autoantibody quantity alone, given samples with similar binding but differential phagocystosis.

### Time from relapse correlates better with CDC and ADCP than the quantity of MOG-IgG.

**Figure 6:**
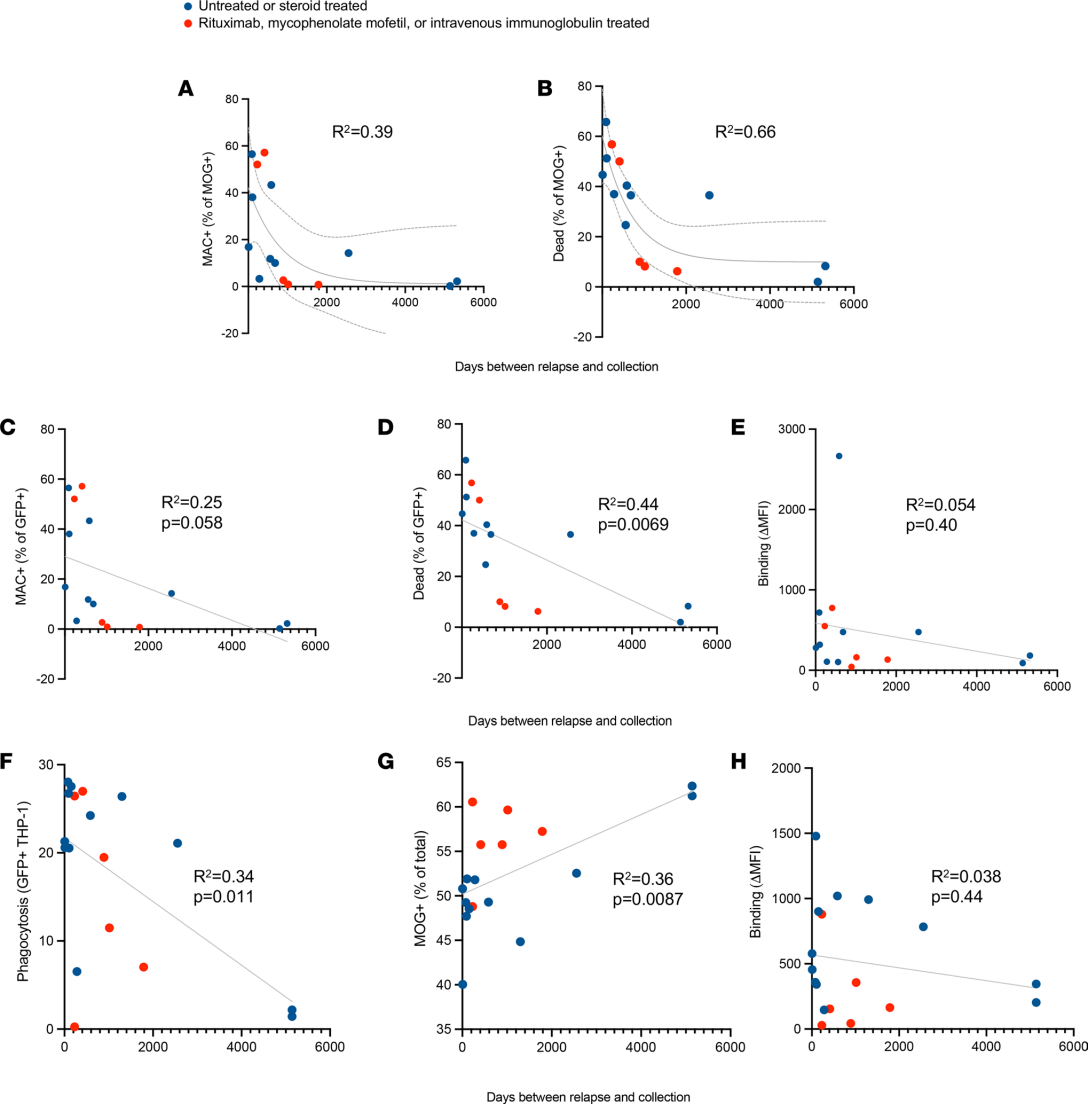
Effector functions better correlate with relapse than do the quantity of MOG-IgG. Regression models were used to assess associations between proximity to relapse and magnitude of CDC, ADCP, and IgG binding to MOG per MOGAD serum sample (*n*_CDC_ = 15, *n*_ADCP_ = 18). (**A** and **B**) MAC formation and dead MOG^+^ cells in CDC assay plotted against days from relapse and fit with exponential decay model (95% CI indicated by dotted lines; goodness of fit, *R*^2^, shown on graphs). (**C** and **D**) MAC formation and dead MOG^+^ cells in CDC assay. (**E**) MOG-IgG binding compared with days from relapse and fit with linear model. (**F** and **G**) Phagocytosing macrophages and percent MOG^+^ cells out of total HEK cells measured in the ADCP assay. (**H**) MOG-IgG binding plotted against days from relapse and fit with linear model. Each dot represents a patient (average of duplicates). For linear models, goodness of fit, *R*^2^, and significance of nonzero slope, *P* value, are shown on graph.

Next, we explored whether serum effector functions correlate with relapse. Relapse dates for 15 of the MOGAD samples used in the CDC assay were available and used to evaluate the association between CDC and the days between most recent prior relapse and sample collection. We compared exponential decay and linear models. First, we found that exponential decay best fit days from relapse versus MAC^+^ and dead MOG^+^ cells with a goodness of fit of *R*^2^ = 0.39 and 0.66, respectively (Figure 6, A and B). Linear regression modeling indicated a reduction in MAC formation further from relapse (*P* = 0.0069) and showed a trend in a reduction in CDC further from relapse (*P* = 0.058) (Figure 6, C and D). However, neither linear regression nor exponential decay modeling fit days from relapse versus MOG binding, with *R*^2^ = 0.054 for both (Figure 6E). Moreover, no association was found between these metrics (*P* = 0.40). Considering that patients further from relapse may be undergoing different treatment regimens, we stratified samples that were untreated or undergoing steroid treatment from those undergoing more rigorous treatment, such as rituximab, mycophenolate mofetil, or i.v. immunoglobulin. We observed no differences in CDC (MAC formation or death) in relation to days between relapse and collection based on treatment (*P* ≤ 0.5).

Linear regression modeling of ADCP measured with phagocytosing macrophages or percent change in MOG^+^ cells, versus days from relapse, had a goodness of fit of *R*^2^ = 0.34 and 0.359, respectively, for the 18 MOGAD samples with relapse dates (Figure 6, F and G). ADCP decreased further from relapse, exemplified by reduced phagocytosing macrophages (*P* = 0.011) and loss of MOG^+^ cells (*P* = 0.0087). However, like CDC, regression modeling did not fit binding versus relapse for these samples (*R*^2^ = 0.038), and no association was found between these metrics (*P* = 0.44) (Figure 6H). We observed no differences in phagocytosis (*P* = 0.5), days from relapse (*P* = 0.5), or percent MOG^+^ cells (*P* = 0.071) upon stratification by treatment. Therefore, while our serum cohorts exhibited reduced CDC and ADCP capability further from relapse, we did not observe a correlation between the quantity of MOG-IgG and relapse. This exemplifies the potential elevated cytotoxic capabilities of MOG-IgG closer to disease manifestation.

### MOGAD serum initiates CA when cell death is experimentally prevented.

We considered the possibility that death of MOG^+^ cells in the CDC assay might result in the underestimation of CA. Thus, we wished to evaluate whether a more sensitive assay could be designed by preventing completion of the complement cascade.

**Figure 7:**
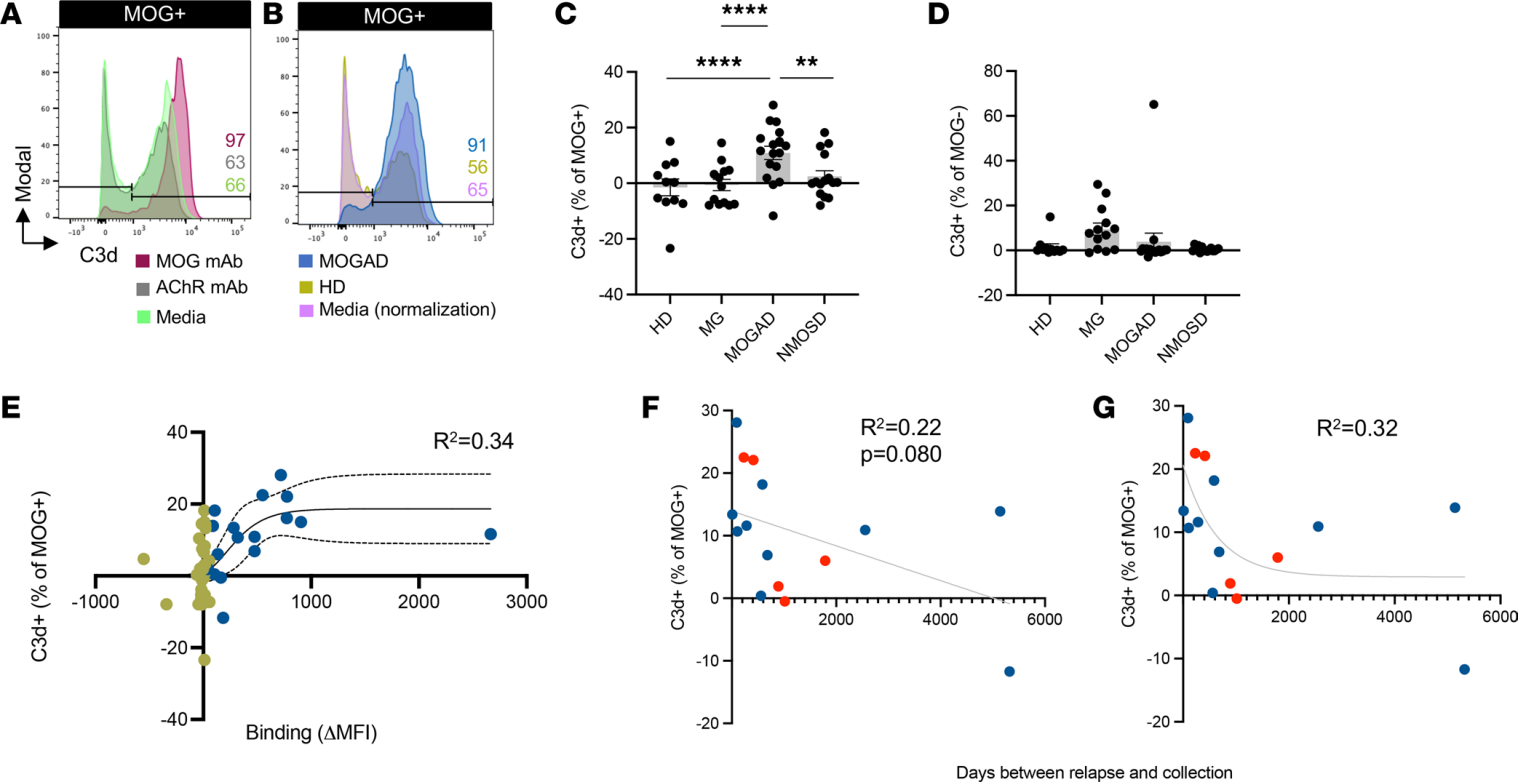
MOGAD patient serum induces CA on live MOG-expressing cells. The CA assay utilizes C8-depleted NHS as the complement source to prevent MAC formation and CDC. Thus, C3d deposition can be monitored without loss of MOG^+^ cells. (**A**) Histograms depict C3d deposition on MOG^+^ cells in the presence of 1 μg/mL MOG mAb in comparison with AChR mAb. Each experiment was performed at least twice in duplicate. Frequencies of indicated gates depicted on plots. (**B**) Representative histograms depict C3d^+^MOG^+^ cells by MOGAD versus HD HI serum. (**C** and **D**) Comparative C3d deposition on (**C**) MOG^+^ and (**D**) MOG^–^ cells by HI serum from patients with MOGAD (*n*_CDC_ = 17), MG (*n*_CDC_ = 13), and NMOSD (*n*_CDC_ = 15) and HD (*n*_CDC_ = 11). Each dot represents a patient (average of duplicates), normalized to media-only control, and bars depict mean ± SEM. Normality test followed by 1-way ANOVA for **C** (*P* = 1.2 ***×*** 10^–3^) and **D** (*P* = 0.12). For *P* ≤ 0.05, multiple comparisons were corrected with FDR of 0.05 and depicted on graph (**P ≤ 0.01, ****P ≤ 0.001). (**E**) C3d^+^MOG^+^ cells upon CA assay versus binding to MOG, fit with Gompertz model (95% cCI indicated by dotted lines; goodness of fit, *R*^2^, shown on graph). (**F** and **G**) C3d deposition on MOG^+^ cells in CA assay plotted against days from relapse for MOGAD samples (*n*_CDC_ = 15) and fit with (**F**) linear model and (**G**) exponential decay model (goodness of fit, *R*^2^, shown on graphs; significance of nonzero slope, *P* value, is shown for linear model).

Thus, we employed NHS depleted of C8, a requirement for MAC formation, as a source of human complement. Then, we measured CA using an antibody specific for C3d (47), which covalently attaches to target cells upon complement initiation. We observed elevated C3d deposition on MOG^+^ cells in the presence of MOG mAb or MOGAD patient serum in comparison with AChR mAb or HD serum, respectively (Figure 7, A and B), indicating that MOG autoantibody–mediated CA had occurred. Moreover, death of MOG^+^ cells was not detected by the MOG or AChR mAbs in the CA assay, despite C3d deposition, indicating effective abrogation of CDC (Supplemental Figure 4, A–C). However, a sizable population (over half) of the MOG^+^ cells exhibited C3d deposition in the presence of negative controls, including AChR mAb, no antibody source (media alone), or HD serum. This implies nonspecific or autoantibody-independent C3d deposition or C3d antibody binding.

Collectively, MOGAD serum (Table 1 and Supplemental Table 1) resulted in a mean 11% C3d^+^MOG^+^ cells (normalized to media alone), greater than that of HD (mean, –1.5%), MG (mean, –0.62%), and NMOSD (mean, 2.5%) (Figure 7C). CA was specific to MOG, as there was no difference in frequency of C3d^+^MOG^–^ cells (Figure 7D; *P* = 0.12). However, this assay exhibited an SNR of MOGAD/HD serum of 1.0. We observed that the same MOGAD outlier that induced MAC formation and death of MOG^–^ cells caused elevated C3d deposition on MOG^–^ cells of 65%. A direct comparison of CA and CDC induction showed that 3 MOGAD samples exhibited an elevated frequency of C3d^+^ over MAC^+^MOG^+^ cells, while the rest induced a similar or elevated MAC formation compared with C3d (Supplemental Figure 4D). Linear modeling of C3d deposition versus MAC formation indicated a positive correlation (*P* = 0.0024; Supplemental Figure 4E). Like the CDC assay metrics, the correlation between C3d and binding was best modeled by the Gompertz curve, but the fit was not as good (*R*^2^ = 0.34) (Figure 7E). However, linear regression showed a positive association between binding and C3d (*P* = 0.0015). Both linear regression and exponential decay fit days from relapse versus C3d poorly (*R*^2^ < 0.35), and linear regression did not depict an association (*P* = 0.08) (Figure 7, F and G). Therefore, MAC formation and CDC correlate better with the quantity of MOG autoantibodies and days from relapse than does C3d deposition.

Given that 1 MOGAD sample induced CDC and CA of both MOG^+^ and MOG^–^ cells, we explored whether this sample exhibited nonspecific IgG reactivity; however, this sample did not exhibit significant IgG binding to MOG^–^ cells (Supplemental Figure 5A, red arrow). This sample also induced CDC of AQP4^+^ and AQP4^–^ cells (not shown). Finally, we performed a MOG CBA, using an anti-IgM secondary antibody rather than anti-IgG. In this assay, we found that this sample harbored significant IgM binding to MOG^–^ cells, suggesting direct binding to the HEK cells (Supplemental Figure 5, B and C; red arrows); this explains the nonspecific CA and CDC of HEK cells. This patient had not been diagnosed with other autoimmune conditions, and no other explanatory clinical or demographic characteristics were identified. Of note, 1 other sample exhibited high MOG^–^ IgG and also MOG^+^ IgM (Supplemental Figure 5, A and C, blue arrows); this is the sample in the aforementioned assays with the highest MOG-IgG ΔMFI (Figure 5), and it induced the second highest nonspecific death of MOG^–^ cells in the CDC assay (Figure 3F).

### MOGAD serum induces ADCC of MOG-expressing cells.

**Figure 8:**
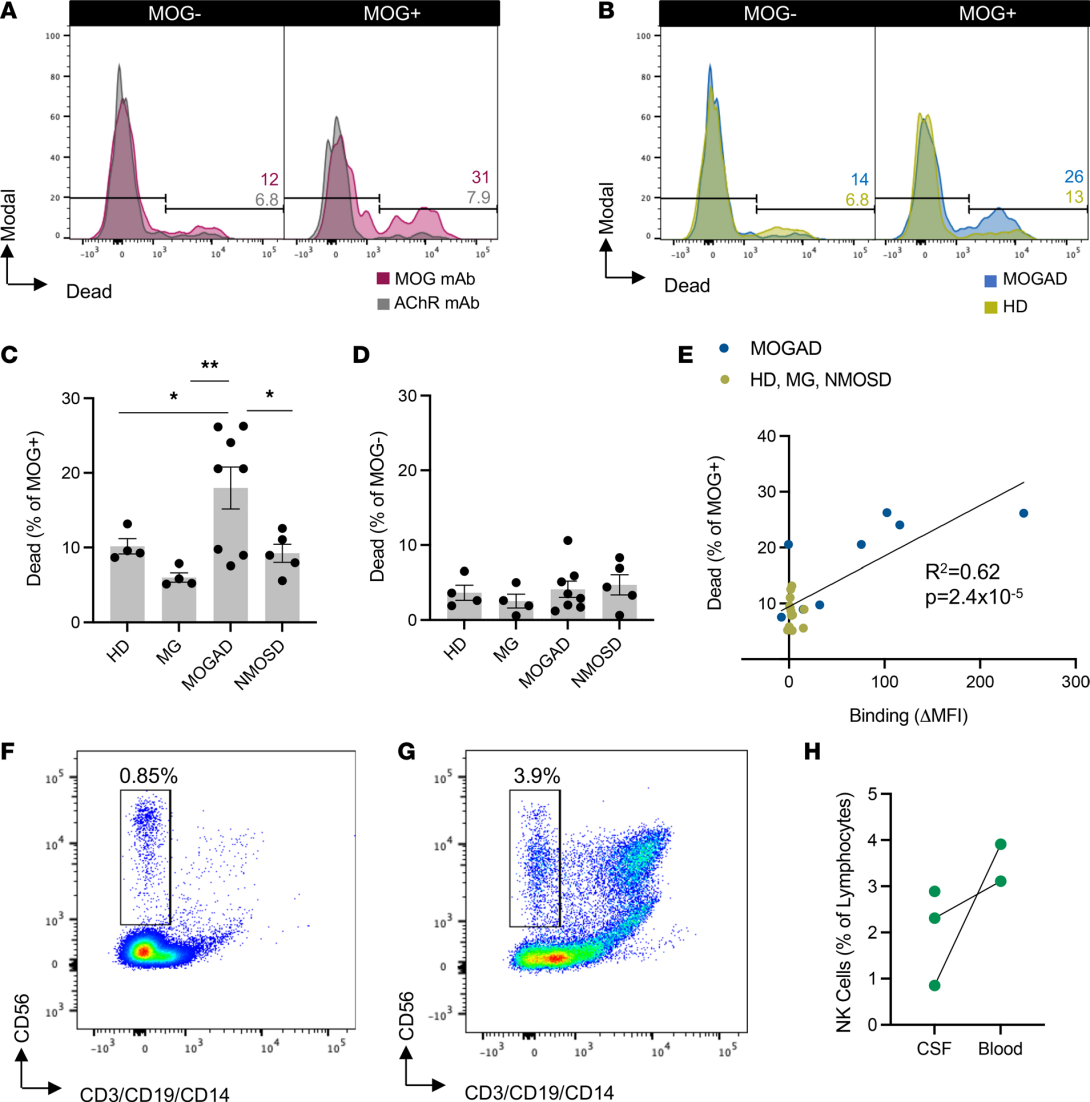
MOGAD patient serum induces ADCC of live MOG-expressing cells. The ADCC assay was performed similarly to the ADCP assay with pooled HD NK cells to mediate cytotoxicity rather than macrophages for phagocytosis. A live/dead stain was used to identify killed HEK cells. (**A**) Histograms depict dead MOG^–^ and MOG^+^ cells with 1 μg/mL MOG versus AChR mAb in the ADCC assay. Each experiment was performed at least 3 times in duplicate. Frequencies of indicated gates depicted on plots. (**B**) Representative histograms depict dead MOG^–^ and MOG^+^ cells by HI MOGAD versus HD serum. (**C** and **D**) Comparative ADCC of (**C**) MOG^+^ and (**D**) MOG^–^ cells by HI serum from patients with MOGAD (*n*_ADCC_ = 8), MG (*n*_ADCC_ = 4), and NMOSD (*n*_ADCC_ = 5) and HD (*n*_ADCC_ = 4). Each dot represents a patient (average of duplicates), and bars depict mean ± SEM. Normality test followed by 1-way ANOVA for **C** (*P* = 0.0075) and **D** (*P* = 0.68). For *P* ≤ 0.05, multiple comparisons were corrected with FDR of 0.05 and depicted on graph (*P ≤ 0.05, **P ≤ 0.01). (**E**) Frequency of MOG^+^ cells out of total HEK in the ADCC assay versus IgG binding to MOG, fit with linear regression model (goodness of fit, *R*^2^, and significance of nonzero slope, *P* value, shown on graph). Flow cytometry was then used to identify the presence of NK cells (CD56^+^CD3^–^CD19^–^CD14^–^ lymphocytes) in the CSF of 3 relapsing patients with MOGAD. (**F** and **G**) Representative gating of NK cells out of lymphocytes in (**F**) CSF and (**G**) blood from 1 patient. (**H**) Frequency of NK cells out of lymphocytes in CSF versus blood in patients with MOGAD.

While histologic studies have not yet identified NK cells — mediators of ADCC — in MOGAD lesions, MOG-IgG from a cohort of pediatric patients were shown to mediate ADCC (33). Thus, we sought to evaluate ADCC as an additional pathogenic mechanism mediated by MOGAD patient autoantibodies by developing a MOG ADCC assay combining the CBA and established flow cytometry ADCC assays (48, 49). This assay only differed from the ADCP assay in that pooled HD NK cells were utilized as the effector source rather than macrophages. Then, HEK cell death was evaluated using a live/dead stain. We observed ADCC of MOG^+^ cells in the presence of MOG mAb but not in the presence of an AChR mAb (Figure 8A). We analyzed a set of specimens composed of 8 MOGAD patient serum samples and 13 control serum samples, including HD and patients with MG and NMOSD (Table 4 and Supplemental Table 1). We observed NK cell–mediated ADCC of MOG^+^ cells but not MOG^–^ cells, resulting from incubation with MOGAD patient serum (Figure 8, B and C). In particular, MOGAD serum resulted in a mean 18% MOG^+^ cell death, significantly more than HD (mean 10%), MG (mean 6.0%), and NMOSD (mean 9.2%). There was no difference in the frequency of MOG^–^ cell death between groups (Figure 8D). Importantly, not all MOGAD samples mediated cell death, similar to what we observed in the CDC assay. Three of the 8 (38%) MOGAD samples resulted in less than 10% cell death. The SNR of MOGAD/HD serum ADCC was 4.2. Linear regression indicated that samples with greater MOG-binding IgG induced greater death of MOG^+^ cells, suggesting that increased autoantibody binding to MOG is positively associated with ADCC (*R*^2^ = 0.62, *P* = 0.000024; Figure 8E).

In order to evaluate whether NK cells migrate to the CNS in MOGAD, we performed flow cytometry on fresh cerebrospinal fluid (CSF) from 3 patients with MOGAD during a relapse (Supplemental Table 1). NK cells, defined as CD56^+^CD3^–^CD19^–^CD14^–^ lymphocytes, were distinctly detected in all 3 CSF samples (Figure 8, F–H). Fresh peripheral blood mononuclear cell (PBMC) samples were available for 2 of these patients; both showed the presence of NK cells at slightly higher frequencies than in the CSF. Differences in the frequency of NK cells in the CSF and blood, lack of RBCs in CSF, as well as skewing of phenotype (higher CD56 expression by CSF NK cells) suggest that the presence of NK cells in the CSF is not a product of blood contamination. Given the presence of NK cells intrathecally and the demonstrated ADCC capability of MOGAD serum autoantibodies, it is possible that NK cells contribute to damage of MOG-expressing cells through ADCC in patients.

## Discussion

Here, we developed high-throughput assays to measure CDC, CA, ADCP, and ADCC of live MOG-expressing cells. Importantly, these cells express full-length human MOG in its native conformation. With these assays, we confirmed that MOGAD patient autoantibodies have cytotoxic capacity through these effector functions. Notably, we observed that (a) cytotoxicity was not incumbent on MOG autoantibody quantity alone, although there is a positive correlation; (b) engagement of effector functions by MOGAD patient serum is bimodal, with some patients’ sera exhibiting cytotoxic capacity while others did not; (c) the magnitude of CDC and ADCP is increased at time points closer to relapse, while MOG-IgG concentration was not; (d) it is possible for all IgG subclasses to mediate damage to MOG-expressing cells; and (e) these activities can be observed in both the CNS and periphery linking the in vitro assays to disease pathology. These collective data identify pathways that may underlie the clinical manifestations of MOGAD and represent areas potentially ripe for therapeutic intervention. This work suggests a possible utility of effector function assays for relapse prediction, although more work is needed to fully explore the temporal patterns of MOG-IgG–associated cytotoxicity and to test predictive value.

Intriguingly, like relapse itself, MOGAD patient serum engagement of effector functions appears binary. MOGAD serum either induced an effector function or it did not. While we observed variation within those groups, there was limited continuity between them. Thus, we investigated whether clinical characteristics, particularly treatment status, MOG-IgG magnitude, and days from relapse, correlated with induction of effector functions. While several retrospective observational studies have reported the annual relapse rate to be reduced in patients with MOGAD treated with rituximab or even more so with i.v. immunoglobulin (50–56), for unclear reasons, rituximab appears to be less effective at preventing attacks in MOGAD than NMOSD (57). Indeed, our cohort does not show a difference in autoantibody-mediated effector functions in relation to treatment, and more strikingly, while we found correlations between MOG-IgG binding and effector function and between effector function and days from relapse, we did not find a correlation between MOG-IgG binding and days from relapse. This indicates the involvement of other factors, such as autoantibody characteristics (epitope specificity, glycosylation, subclass, affinity) or presence of effector molecules or cells in relapse. Indeed, a recent study shows that effector function–enhancing glycosylation of patient antibodies is elevated closer to relapse in MOGAD (58).

Thus, while rituximab and i.v. immunoglobulin are aimed at reducing the concentration of pathogenic autoantibodies, additional studies are required to understand if this is sufficient to prevent relapse or if effector functions should also be modulated. For example, further clinical studies would be required to investigate the efficacy of complement inhibitors. Interpatient CDC differences, as shown here, imply that complement inhibitors may not be equally effective for all patients with MOGAD. We have observed that AChR IgG^+^ MG samples also induce variable MAC formation not solely attributable to the magnitude of AChR binding IgG in the CBA (59). This is significant, considering that complement inhibition by eculizumab is not universally efficacious in MG patients and efficacy is not correlated with AChR autoantibody titer (60–62). Therefore, assays to measure CDC and other cellular effector functions may help predict which patients are most likely to respond to complement inhibitors and other therapies. Only minor modifications to the live MOG-binding CBA, the current clinical standard for the assessment of MOG autoantibodies in patient serum, are required for these high-throughput effector function assays. These additions include a complement source and a live/dead stain and/or complement antibody for the CDC/CA assays, NK cells and a live/dead stain for the ADCC assay, and macrophages for the ADCP assay. These assays offer ease-of-use and high-throughput feasibility for quick adoption into clinical practice and, therefore, have the potential to aid in personalized treatment, prognosis, or relapse prediction.

The relationship between CDC and MOG-IgG exhibits precipitous changes at specific concentrations. These observations suggest that CDC occurs in a stepwise manner based on a threshold, as described by the Gompertz model, rather than continuously increasing. We speculate that this reflects that a certain amount of antibody is required to facilitate optimal Fc-Fc interaction and efficient activation of the complement cascade and other Fc-dependent pathogenic mechanisms, as has been elegantly shown with AQP4 autoantibodies (63). While the optimal geometry of MOG for autoantibody-mediated pathology has yet to be fully understood, the cytosolic tail seems integral to multimerization, considering enhanced binding of patient autoantibodies (64, 65). ADCP induction did not appear to share this requirement and suggests the possibility of a more linear relationship between binding and phagocytosis: Within the range of concentrations of mAbs tested, a Gompertz curve also known as a Sigmoid or “S” curve, is detected for CDC; in contrast, the majority of these concentrations appear to mediate maximal ADCP. These data may be a reflection of the receptor-mediated interaction between phagocyte and antibody in the latter. While C1q, the initiating complement protein, has 6 Fc binding sites, each Fc receptor has 1 or 2 (66–69). Therefore, the initial barrier for C1q binding, requiring a certain threshold of antibody bound to cells and their organization for Fc interaction, may not be required for ADCP. Considering that all IgG can also mediate ADCP, it is worth considering the possible contribution of ADCP to tissue damage.

Previous histologic studies of MOGAD patient lesions have shown MOG-laden microglia and macrophages within demyelinating lesions (28, 37). Whether their activity is purely janitorial or cytotoxic has not been clarified. Our data suggest that it is possible that ADCP is occurring within lesions. In the presence of MOG autoantibody, macrophages phagocytose MOG-expressing cells, resulting in a depletion of these MOG-expressing cells. Our paired data presenting an individual with MOG-laden macrophages as well as serum autoantibodies capable of ADCP strengthen this hypothesis. However, the relative contributions of ADCC and NK cells are more difficult to identify in histology because (a) they are innate cells that are typically present early in the inflammatory response, (b) they consist of a small population of lymphocytes (2%–5% in blood), and (c) their phenotype changes upon cytotoxic activity (70–73). While the contribution of ADCC to MOGAD pathogenicity is still unclear, the lack of histologic evidence does not preclude this possibility. As shown, NK cells were distinctly identified in 3 of 3 MOGAD CSF samples collected during relapse. Patients with MS also exhibit the presence of NK cells in CSF, at approximately 3% of lymphocytes, as well as in demyelinating lesions (74–77). Their presence in the CSF appears to be elevated during active disease versus stable remission (75). Given ADCC capability of human MOGAD serum autoantibodies and the presence of NK cells in MOGAD patient CSF, their contribution remains possible. It is also possible that their contributions to lesion formation is minor in comparison with CDC and/or ADCP, but their role in disease may not be limited to cytotoxic activity. Considering their ability to assume HLA-DR expression and antigen presentation–like DCs (78–80), it is also possible that NK cells perpetuate the adaptive immune response in patients with MOGAD through antigen presentation. Ultimately, further study is required to gain a complete understanding of the pathomechanisms of MOGAD and their relative and cumulative clinical impact.

We note several limitations to the present study. First, cell-based in vitro assays cannot recapitulate the organization of the CNS microenvironment, conformation of myelin, relative dynamic temporal quantities of immune cells and components present in the parenchyma, or endogenous expression of inhibitory and activating ligands on myelin and other neural cells. Nonetheless, our demonstration of CDC and ADCP in serum samples from a patient with MOGAD who had complement deposition and macrophage/microglial activation on brain biopsy provides a link to the CNS microenvironment. Considering the challenge of studying MOGAD pathogenesis in animal models due to limited binding of human MOG autoantibodies to rodent MOG (35) and differences in human versus murine IgG subclasses, FcR, their association, and effector functions (81–83), in vitro studies using human MOG are a useful complementary tool for attaining a better understanding of the disease and identifying effective biomarkers and therapeutics. Our work supports the utility of such assays by showing congruence with patient CNS neuropathology. Cohorts of longitudinal serum biospecimens and those allowing for greater stratification based on treatment and other clinical characteristics will be useful in extending and confirming our findings.

In summary, we found that autoantibodies from patients with MOGAD are capable of damaging live MOG-expressing cells through at least 3 effector functions. Importantly, we show that MOG autoantibodies are capable of mediating ADCP, suggesting the possibility that macrophages and microglia in demyelinating lesions contribute to pathology. Moreover, we found a correlation between CDC and ADCP with CNS neuropathology and relapse. Notably, we also found ADCC effector cells, NK cells, in the CSF of patients with MOGAD during relapse. Thus, we confirm and extend prior findings regarding the effector functions of MOG autoantibodies, with the addition of ADCP, and we also show the potential clinical utility of in vitro effector function assays.

## Methods

### CBA.

All CBA utilized live cells expressing full-length human MOG in its native conformation, as previously described (49). In brief, HEK cells were transiently transfected with 18 μg MOG-GFP plasmid using branched polyethylenimine. The MOG-GFP expression vector contains full-length human MOG in a pEGFP-N plasmid vector provided by Markus Reindl (Medical University of Innsbruck, Innsbruck, Austria). After 16 hours, the cells were washed and incubated for 24 hours. Our protocol produces 50%–60% transfection efficiency.

For the CBA, these cells were trypsinized, washed, and plated at 25,000 cells per well of 96-well U-bottom plate. Following this, the cells were incubated with mAbs (human anti–AChR IgG1 antibody, called mAb 637, and a murine-derived anti-MOG mAb engineered for expression with human IgG1 and κ constant regions, called mAb 8-18C5; refs. 84, 85) or patient serum (1:10 dilution) for 1 hour, with shaking, at 4°C. The cells were then washed and incubated with 1:1,000 Alexa Fluor 647–conjugated rabbit anti–human IgG Fcγ fragment (309-605-008, Jackson ImmunoResearch Laboratories) for 30 minutes, with shaking, at 4°C. To detect IgM, we used 1:1,000 DyLight 405–conjugated goat anti–human IgM Fc5μ fragment (109-475-043, Jackson ImmunoResearch Laboratories). Then, the cells were washed and resuspended with FACS buffer and analyzed using a BD LSR Fortessa flow cytometer.

Single, live transfected MOG-expressing cells were measured in the GFP channel (MOG^+^), while those that did not express MOG-GFP were utilized as a negative control (MOG^–^). IgG binding was measured in the Alexa Fluor 647 channel, and IgM binding was measured in the Cascade Blue channel. CBA results were presented as the difference in IgG (Alexa Fluor 647) MFI between MOG^+^ and MOG^–^ cells, called ΔMFI. Samples were run on the CBA in duplicate, and results are presented as their mean. All study assays were run with positive and negative control mAbs (MOG and AChR, respectively).

### MOG mAb Fc vector subcloning.

IgG2, IgG3, and IgG4 expression vectors were engineered from our human IgG1 heavy chain expression vector (86) using published human constant regions (GenBank; AXN93670.2, AK097307.1, KJ901516.1) to replace that of IgG1. An expression vector with a mutated Fc region shown to abrogate effector functions was also engineered by introducing L234A, L235E, G237A, K322A, and P331S mutations described for pRVL-6 (40) into our IgG1 heavy-chain expression vector (FcMt). The sequence integrity of these new heavy-chain expression vectors was confirmed by both Sanger sequencing of the insert and sequencing of the entire plasmid with the Oxford Nanopore platform (Plasmidsaurus). The heavy-chain variable region of the MOG mAb was subcloned into the IgG2, IgG3, IgG4, and FcMt expression vectors at the AfeI and ApaI sites. After confirming the sequences, the plasmids were transformed into NEB 5-α competent *E. coli* (New England BioLabs). Plasmid DNA was isolated with the QIAprep Spin Miniprep Kit (Qiagen) and sequenced by Sanger sequencing to confirm the presence of each specific variable region. These heavy chains were expressed along with the MOG mAb light chain in HEK293A cells. The antibodies were purified using Protein G Sepharose (Cytiva). Microvolume spectrophotometry UV_280 nm_ was used to quantify concentration. To verify human IgG subclass expression, ELISA plates were coated with subclass specific antibodies for IgG1 (MH1015, Thermo Fisher Scientific), IgG2 (05-3500, Thermo Fisher Scientific), IgG3 (05-3600, Thermo Fisher Scientific), IgG4 (MA5-16716, Thermo Fisher Scientific), and total IgG (109-005-098, Jackson ImmunoResearch) at 5 μg/mL (250 ng/well), incubated with serial dilutions of the mAbs from 0.0046 to 10 μg/mL, and detected with a peroxidase-conjugated goat anti–human IgG antibody (109-035-098, Jackson ImmunoResearch, 1:20,000). This was followed by standard procedure for colorimetric development, detection, and analysis.

### Autoantibody-mediated complement assays.

The CDC assay was modified from the aforementioned MOG CBA and previously reported CDC assays (19, 59). In brief, sera (1:10) or mAbs (1μg/mL unless otherwise indicated) were added to cells in 96-well round-bottom plates and incubated for 15 minutes at room temperature. All serum samples were HI prior to use to inactivate endogenous complement proteins. Then, NHS or factor B–depleted NHS (Comptech) was added as a complement source (10% final concentration). The plates were incubated at 37°C and shaken intermittently. After the designated incubation period, the cells were washed and incubated with Near IR Live/Dead (Invitrogen) stain for 30 minutes on ice. Then, they were washed again before incubation with 1:50 anti-C9neo (mAb aE11, Hycult Biotech) on ice for 30 minutes to identify MAC formation. After another wash, the cells were incubated with 1:1,000 PE-conjugated anti–mouse IgG2A (RMG2a-62, BioLegend) for 30 minutes on ice. Finally, the cells were washed again and prepared for analysis on a BD LSR Fortessa flow cytometer. MOG^+^ and MOG^–^ cells were detected as in the CBA, and dead cells were measured in the APC-Cy7 channel and MAC^+^ cells in the PE channel. Data were normalized by subtracting the mean media-only conditions (no antibodies or serum).

For the CA assay, C8-depleted NHS (Comptech) was used (10% final concentration) instead of NHS. After the incubation period, the cells were immunolabeled with 1:1,000 mouse anti–C3d IgG1 (47), provided by Joshua M. Thurman, Kelly Fahnoe, and Stefan Wawersik of Q32 Bio (Waltham, Massachusetts, USA). Then, they were incubated with 1:1,000 PE-Cy7 anti–mouse IgG1 antibody (RMG1-1, BioLegend). Both incubations occurred on ice for 30 minutes. The cells were prepared and analyzed as in the CDC assay. MOG^+^ and MOG^–^ cells were detected as in the CBA, and C3d^+^ cells were measured in the PE-Cy7 channel. Normalized data are presented by subtracting mean media-only condition (no antibodies or serum).

### Autoantibody-mediated ADCC and ADCP assays.

The ADCC and ADCP assays are also modifications of the CBA in combination with previously established flow cytometry-based ADCC (48) and ADCP assays (87, 88). In this case, serum (1:10) or mAbs (1μg/mL unless otherwise indicated) were added to 5,000 MOG-GFP transfected HEK cells plated in 96-well round-bottom plates and incubated for 15 minutes at room temperature. All serum samples were HI prior to use to inactivate endogenous complement proteins. Then, effector cells were added at a 10:1 effector/target ratio. For the ADCC assay, the effector cells were NK cells that were magnetically isolated (EasySep Human NK Cell Isolation Kit, STEMCELL Technologies) from pooled HD-derived cryopreserved PBMCs. The effector cells for the ADCP assay consisted of the THP-1 macrophage cell line (ATCC), labeled with CellTrace Violet (Thermo Fisher Scientific). After addition of the effector cells, the plates were then incubated at 37°C for 4 hours and shaken intermittently. For the ADCC assay, the cells were then stained with Near IR Live/Dead (Invitrogen) stain for 30 minutes on ice, to identify killed target cells. MOG^+^ and MOG^–^ were detected as in the CBA as GFP^+^. For the ADCP assay, macrophages were identified in the V450 channel and phagocytosis of MOG^+^ cells in the GFP channel. Data were normalized to the mean media-only condition (no antibodies or serum).

### Histology.

IHC was performed on formalin-fixed paraffin-embedded 5 μm–thick sections with EnVisionTM FLEX IHC system (DAKO). Steam antigen retrieval with citric acid buffer (pH 6.0, DAKO) was performed for MAG, MOG, and C9neo staining. Primary antibodies were incubated at 4°C overnight to identify MAG (1:1,000, ab89780, Abcam), MOG (1:1,000, ab109746, Abcam), PLP (1:500, MCA839G, Serotec), C9neo (1:200, monoclonal B7 and polyclonal, from Paul Morgan, Cardiff, United Kingdom), and CD68 (1:100, M0814, DAKO).

### Immunophenotyping.

Fresh CSF cells and PBMCs were isolated and immunophenotyped within 6 hours of sample collection. Briefly, CSF cells were isolated by centrifugation at room temperature at 500*g* for 10 minutes from whole CSF and washed with PBS. PBMCs were isolated by layering whole blood on Ficoll-Paque PLUS (Cytiva), followed by centrifugation at room temperature at 1,000*g* for 25 minutes with minimal acceleration and deceleration, harvesting, and washing. CSF cells and PBMCs were then incubated with LIVE/DEAD Fixable Near-IR Dead Cell Stain (L34975, Invitrogen), anti–CD3-V500 (561416, BD Biosciences), anti–CD14-V500 (561391, BD Biosciences), anti–CD19-BV510 (562947, BD Biosciences), and anti–CD56-APC (362503, BioLegend) for 30 minutes on ice. After washing, they were analyzed on a BD LSR Fortessa flow cytometer. NK cell frequency was calculated as CD56^+^CD3^–^CD19^–^CD14^–^ out of total live lymphocyte singlets.

### Statistics.

Flow cytometry data were analyzed using FlowJo v10 (BD Biosciences). Statistical analyses and regression modeling were performed using GraphPad Prism Version 9. Throughout, *P* ≤ 0.05 was considered significant. Normality was tested for all comparisons using the D’Agostino Pearson omnibus. When multiple normal or nonparametric groups were compared, ordinary 1-way ANOVA or Kruskal-Wallis test was used, respectively. If significant, they were followed by multiple comparisons corrected with a FDR of 0.05 using the Benjamini, Krieger, and Yekutieli method. SNR is calculated as the amplitude of MOGAD serum effector function divided by that of HD (SNR = amplitude_MOGAD_/amplitude_HD_). Amplitude refers to the highest value minus the lowest. Regression models were fit based on least-squares method and compared with one another using AICc.

### Study approval.

This study was approved by the Yale University IRB. Informed written consent according to the Declaration of Helsinki was received from all participants or parents (for minors) prior to inclusion in this study. MOGAD, NMOSD, and HD serum samples, with the exception of the neuropathology case, were collected from the Yale Multiple Sclerosis Clinic, while MG serum samples were collected from the Yale Myasthenia Gravis Clinic (NCT03792659). The MOGAD neuropathology case was identified through the CNS inflammatory demyelinating disease pathologic biobank of the Mayo Clinic (Rochester, Minnesota, USA). All samples selected for this study were from patients with established diagnoses, based on a typical clinical syndrome in conjunction with positive MOG-IgG serostatus in a live CBA with full-length human MOG. Participants provided age and sex information; clinicians/investigators provided all other clinical information. Ages are presented as ranges for patient privacy.

## Author contributions

SSY and KCO designed the study, interpreted the data, and wrote the manuscript. SSY and BF performed experiments, acquired data, and analyzed data. SSY designed and validated methods. AHO consulted on CDC and CA assay design. NL assisted in clinical data acquisition. JMT provided reagents. YG, CFL, and EPF performed experiments, acquired and analyzed data, provided study samples and clinical data, and assisted in writing. NM, RJN, and EEL provided study samples and clinical data. EEL assisted in interpretation and writing. All authors reviewed and edited the manuscript.

## Supplementary Material

Supplemental data

## Figures and Tables

**Table 1:**
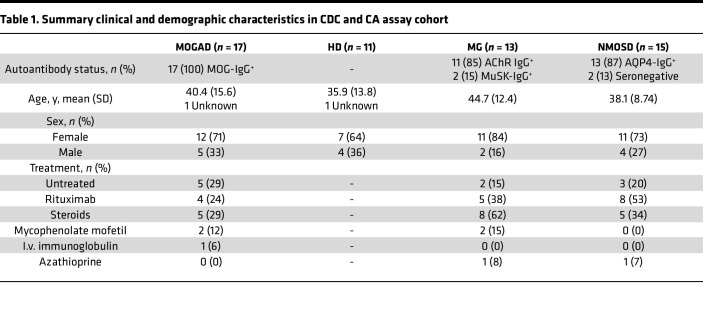
Summary clinical and demographic characteristics in CDC and CA assay cohort

**Table 2:**
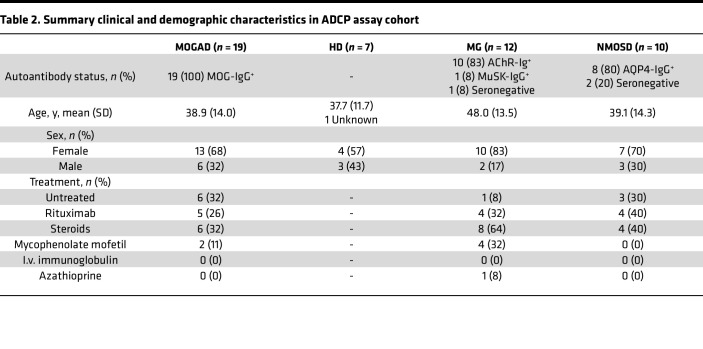
Summary clinical and demographic characteristics in ADCP assay cohort

**Table 3:**
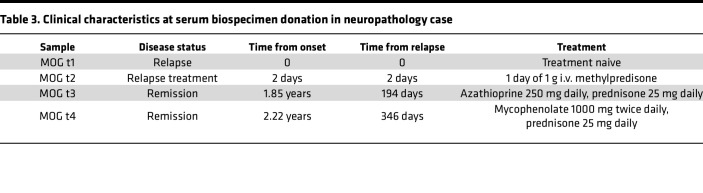
Clinical characteristics at serum biospecimen donation in neuropathology case

**Table 4:**
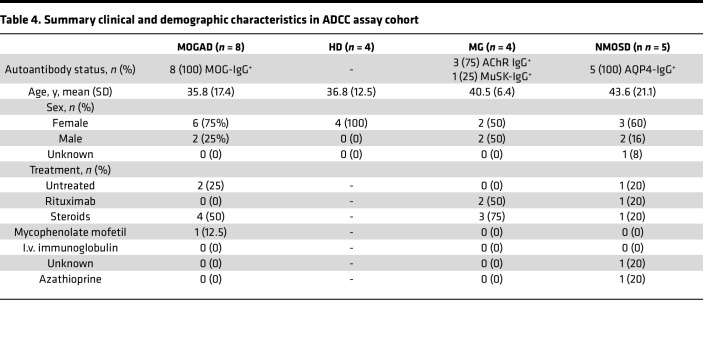
Summary clinical and demographic characteristics in ADCC assay cohort

## Version 1 04/25/2023

In-Press Preview

## Version 2 06/08/2023

Electronic publication
