# Supplementary material for: MOGAD patient autoantibodies induce complement, phagocytosis, and cellular cytotoxicity
Source: JCI Insight. 2023 Jun 8;8(11):e165373. doi: 10.1172/jci.insight.165373 (PMC10393237; doi:10.1172/jci.insight.165373)
Supplement: Supplemental data [file jciinsight-8-165373-s093.pdf]

MOGAD patient autoantibodies induce complement, phagocytosis, and cellular cytotoxicity

Soumya S. Yandamuri, Beata Filipek, Abeer H. Obaid, Nikhil Lele, Joshua M. Thurman, Naila Makhani, Richard J. Nowak, Yong Guo, Claudia F. Lucchinetti, Eoin P. Flanagan, Erin E. Longbrake, and Kevin C. O'Connor

Contents:

Supplementary Table 1. MOGAD patient demographic and clinical characteristics
Supplementary Figure 1. Complement dependent cytotoxicity of MOG-expressing cells by HD, MG, MOGAD, and NMOSD patient serum without the contribution of the alternative complement pathway and without a human complement source.
Supplementary Figure 2. MRI findings in MOGAD neuropathology case.
Supplementary Figure 3. Regression models of MOG-IgG binding versus CDC and ADCP in MOGAD samples.
Supplementary Figure 4. Comparison of CA and CDC.
Supplementary Figure 5. Additional MOG-CBA analyses.

Supplementary Table 1. MOGAD patient demographic and clinical characteristics

DEID	Sex	Age (Range, years)	Treatment	Assays	Days from Relapse Onset	Clinical Titer (Dilution factor)	EDSS
1	F	10-19	Steroids	ADCC, CDC, ADCP	7	1000	2
2	F	50-29	Steroids	ADCC, CDC, ADCP	2555	100	2
3	F	20-29	Untreated	ADCC, CDC, ADCP	586	1000	0
4	F	50-59	Steroids	ADCC, CDC, ADCP	5140	20	4
5	F	50-59	Mycophenolate mofetil	ADCC, CDC, ADCP	229	1000	2
6	M	20-29	Untreated	ADCC, CDC, ADCP	282	100	1.5
7	M	20-29	Steroids	ADCC, CDC, ADCP	106	100	1.5
8	F	30-35	Untreated	ADCC, CDC	559	N/A	1
9	F	20-29	Untreated	CDC, ADCP	93	1000	0
10	F	N/A	Intravenous immunoglobulin	CDC	N/A	1000	0
11	M	50-59	Rituximab	CDC, ADCP	229	100	6
12	F	50-59	Mycophenolate mofetil	CDC, ADCP	411	1000	2.5
13	F	50-59	Untreated	CDC	680	100	2
14	M	30-39	Rituximab	CDC, ADCP	891	100	N/A
15	F	50-59	Steroids	CDC, ADCP	5140	20	4
16	F	30-39	Rituximab	CDC	1016	40	3
17	M	40-49	Rituximab	CDC, ADCP	1787	100	2
18	F	30-39	Untreated	ADCP, CSF	4	100	6
19	M	40-49	Rituximab	ADCP	N/A	100	2
20	F	10-19	Steroids	ADCP	1016	40	3
21	F	50-59	Untreated	ADCP	153	100	3.5
22	F	0-9	Steroids	ADCP	77	1000	N/A
23	F	20-29	Untreated	ADCP	1299	1000	0
24	M	40-49	Steroids	CSF	22	100	0
25	M	30-39	Untreated	CSF	6	100	3

This table includes all samples used in this study except the neuropathology case (detailed information about this case can be found in the text, Table 3, and Supplementary Figure 2.) Age, treatment, days from relapse, and EDSS are from the time of sample collection. Clinical titer at diagnosis and not necessarily at biospecimen collection. The same samples were used for CDC and CA assays. Abbreviations: DEID (deidentified identification), ADCC (antibody-dependent cellular cytotoxicity), CDC (complement-dependent cytotoxicity), ADCP (antibody-dependent cellular phagocytosis), CSF (cerebrospinal fluid immunophenotyping), EDSS (Kurtzke Expanded Disability Status Scale), N/A (not available).

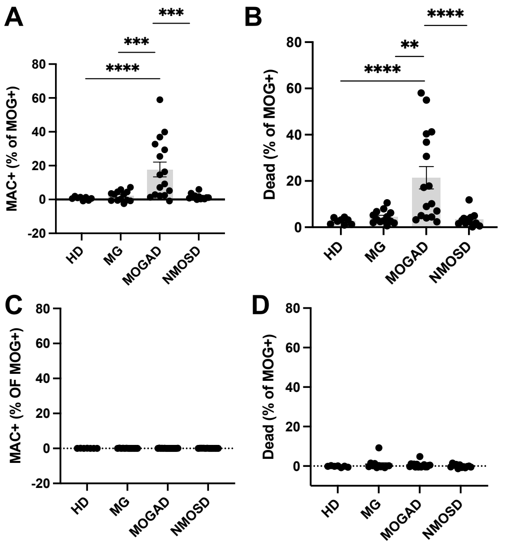

Supplementary Figure 1. Complement dependent cytotoxicity of MOG-expressing cells by HD, MG, MOGAD, and NMOSD patient serum without the contribution of the alternative complement pathway and without a human complement source. (**A**) MAC deposition and (**B**) death of MOG-expressing cells in a CDC assay utilizing Factor B depleted NHS, to abolish the contribution of the alternative complement pathway to CDC. (**C**) MAC deposition and (**D**) death of MOG-expressing cells were quantified in a CDC assay without a complement source such as NHS to evaluate activity of endogenous complement factors in HI donor serum. [Each dot represents a patient (average of duplicates). Each experiment was performed at least twice. Tested for normality, and used Kruskal-Wallis followed by multiple comparisons corrected with false discovery rate of 0.05. Statistics only shown when significant: **$p \leq 0.01$, ***$p \leq 0.005$, ****$p \leq 0.001$.]

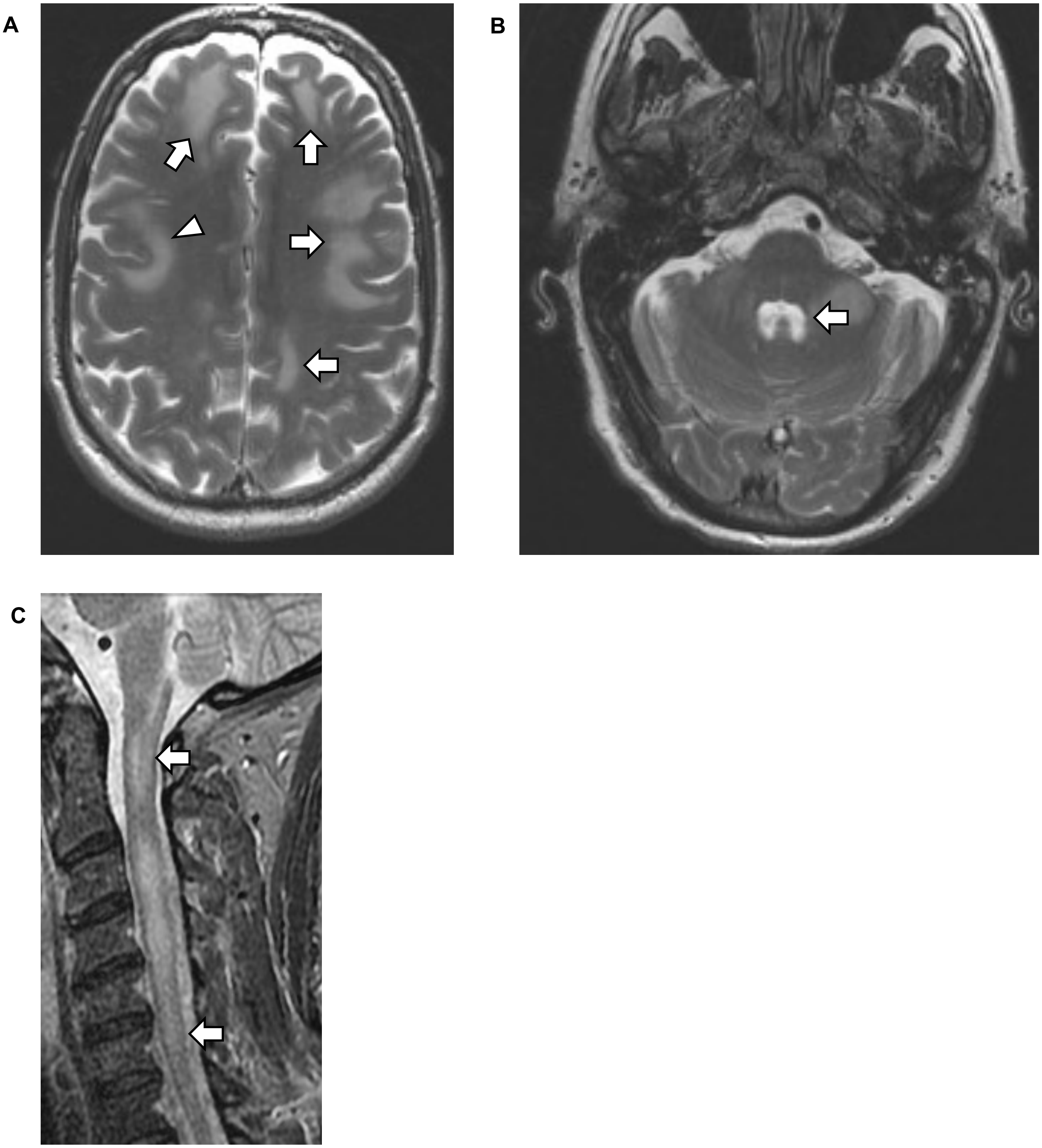

Supplementary Figure 2. Diagnostic findings in MOGAD neuropathology case. Cerebrospinal fluid analysis had revealed a white blood cell count of 139/μL (normal 0-5; 75% lymphocytes), protein of 104 mg/dL (normal range 0-45), and negative oligoclonal bands. Brain and spinal cord MRI revealed multifocal ill-defined (**A**) supratentorial, (**B**) infratentorial, and (**C**) spinal cord T2 hyperintense lesions, indicated by arrows. Biopsy of the right frontal lobe was undertaken (**A**, arrowhead).

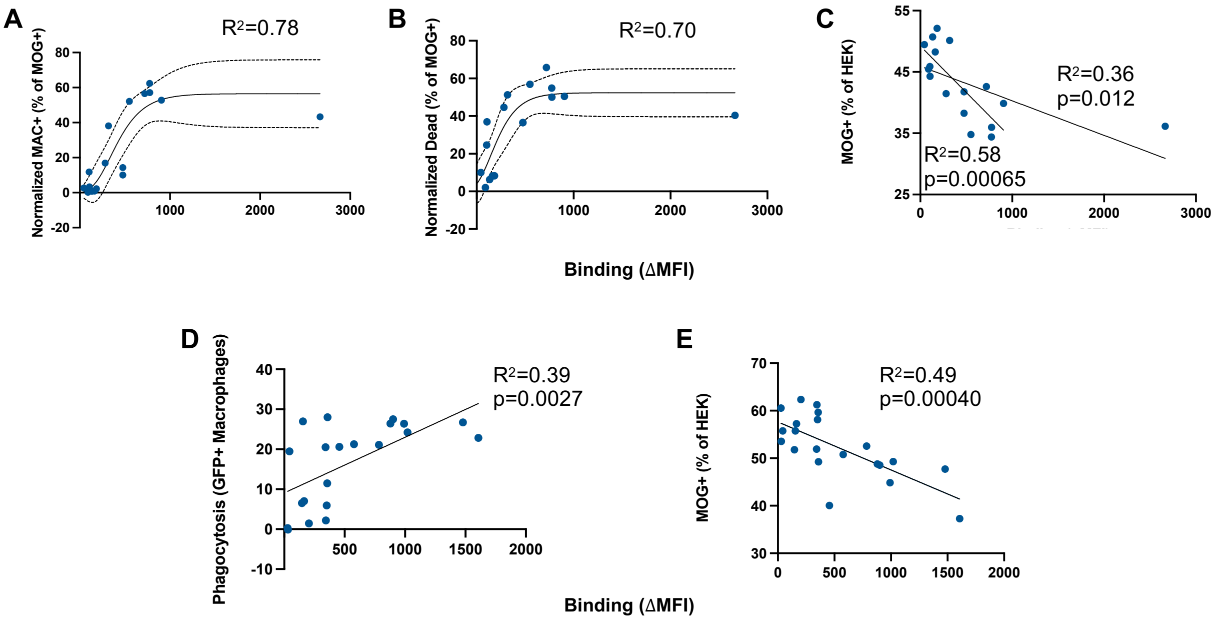

Supplementary Figure 3. Regression models of MOG-IgG binding versus CDC and ADCP in MOGAD samples. Gompertz curve model of (**A**) MAC deposition and (**B**) dead MOG+ cells mediated by MOGAD samples in CDC assay in comparison to sample-specific MOG-binding IgG. (**C**) Linear regression models of percentage of MOG+ cells after CDC by MOGAD samples in comparison to the quantity of MOG-binding IgG, with or without high binding outlier. Linear regression models of (**D**) percent phagocytosis and (**E**) percentage of MOG+ cells after ADCP by MOGAD samples in comparison to the quantity of MOG-binding IgG. [Each dot represents a MOGAD patient (average of duplicates). Goodness of fit (R^2) shown for all models and significance of non-zero slope (p-value), indicating association, shown for linear models.

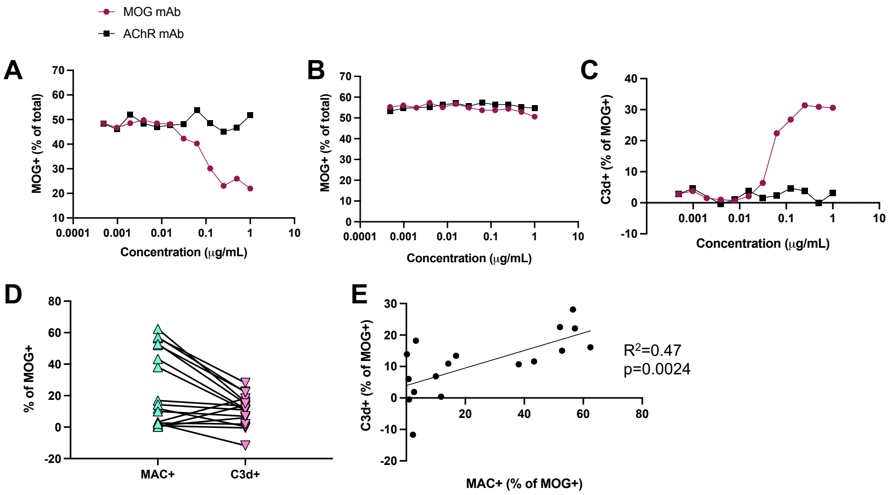

Supplementary Figure 4. Comparison of CA and CDC. (**A**) Percent MOG+ cells out of total HEK cells after CDC assay with serial dilutions of MOG mAb or AChR mAb. (**B**) Percent MOG+ cells out of total HEK cells and (**C**) C3d deposition after CA assay with serial dilutions of MOG mAb or AChR mAb. (**D**) Frequency of MAC formation in CDC assay compared to C3d deposition in CA assay per MOGAD sample. (**E**) Linear regression model of frequency of MAC formation in CDC assay compared to C3d deposition in CA assay per MOGAD sample, with goodness of fit (R^2) and significance of non-zero slope (p-value) shown on graph. [Each experiment was performed at least twice. D, E: Each dot represents a MOGAD patient (average of duplicates).]

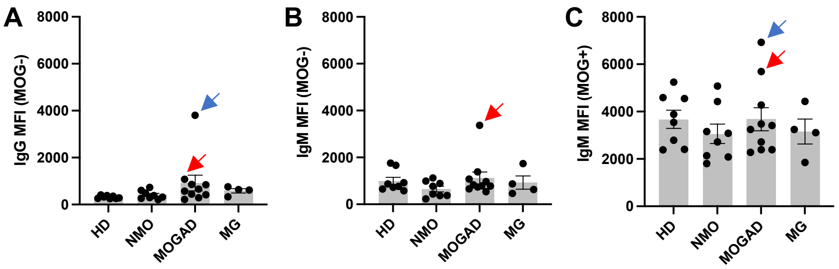

Supplementary Figure 5. Additional MOG-CBA analyses. (**A**) MFI of IgG binding to MOG- cells in MOG-IgG CBA. MFI of IgM binding to (**B**) MOG- and (**C**) MOG+ cells in MOG-IgM CBA. (Blue arrow depicts sample with highest MOG-IgG and red arrow depicts sample producing CDC and CA of MOG- cells). [Each dot represents a patient (average of duplicates). Each experiment was performed at least twice.]
